# Mg/Mn bi-single-atom carbon dot nanozymes with enhanced SOD-like activity and fluorescence for hepatoprotection

**DOI:** 10.1186/s12951-026-04464-5

**Published:** 2026-04-21

**Authors:** Xuejiao Wang, Yu Zhang, Jing Li, Hongjing Cheng, Hanyue Qiu, Jian Jiao, Cui Liu

**Affiliations:** 1 Department of Digestive, China-Japan Union Hospital of Jilin University, Changchun, 130033 P. R. China; 2 Chongqing Key Laboratory of Natural Product Synthesis and Drug Research, Innovative Drug Research Center, School of Pharmaceutical Sciences, Chongqing University, Chongqing, 400044 P. R. China; 3 Department of Pathology, China-Japan Union Hospital of Jilin University, Changchun, 130033 P. R. China

**Keywords:** Acute liver injury, Carbon dots, Bimetallic doped, Nanozyme, SOD-like activity, Fluorescence

## Abstract

Acute liver injury, a critical clinical and public health challenge characterized by rapid hepatocellular dysfunction, is closely associated with ROS-mediated oxidative stress and inflammation. Nanozymes show protective promise *via* ROS scavenging but suffer from low catalytic efficiency and insufficient self-tracing capacity. Herein, we report Mg, Mn co-doped CD (MgMn@CD) nanozymes with ultra-high SOD-like activity (> 20000 U/mg) and satisfactory fluorescence (quantum yield > 16%). Both Mg and Mn are distributed in the CD structure in a single-atom state. Mg doping promotes the conversion of ketone to enol structures in the CD structure, thereby increasing the structural rigidity and improving fluorescence quantum yield. Meanwhile, Mn synergistically enhances SOD-like activity through coordination with ketone structures and nitrogen-containing structures in the CD structure. MgMn@CD nanozymes demonstrate strong hepatoprotective effects against acetaminophen-induced injury by efficiently scavenging reactive oxygen species, restoring mitochondrial function, and suppressing apoptosis, ferroptosis, and inflammation. In vitro studies confirm their antioxidant and cytoprotective effects, while in vivo experiments show dose-dependent improvements in hepatic function, preservation of mitochondrial integrity, and reduction of proinflammatory cytokine levels. With excellent biocompatibility, favorable hepatic tropism, and coordinated regulation of oxidative stress and apoptotic signaling pathways, MgMn@CDs represent a promising nanozyme platform for mitigating acute liver injury.

**Graphical abstract:**

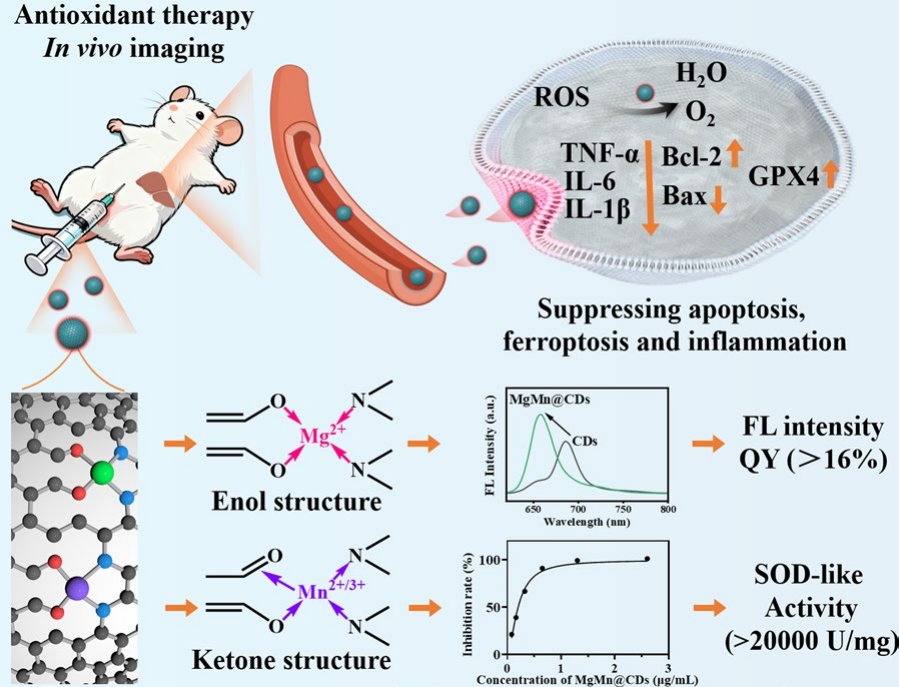

**Supplementary Information:**

The online version contains supplementary material available at 10.1186/s12951-026-04464-5.

## Introduction

Acute liver injury (ALI) is a rapidly progressing condition characterized by significant hepatocellular damage, inflammation, and the potential to progress to hepatic dysfunction [1–3]. A variety of etiologies may contribute to its development, including viral infections (such as hepatitis B and C), drug-induced liver injury, alcoholic liver disease, ischemic events, and autoimmune liver disorders. Although the liver possesses a remarkable regenerative capacity, severe or persistent injury can overwhelm this capacity, leading to acute liver failure, a critical condition associated with high mortality rates [4–7]. Regardless of the initiating factor, ALI involves widespread hepatocellular death accompanied by inflammatory and oxidative stress processes that disrupt hepatic homeostasis [8, 9]. The pathophysiology involves the interplay of hepatocyte apoptosis and necrosis, activation of Kupffer cells and infiltrating immune cells, and excessive release of pro-inflammatory cytokines such as TNF-α, IL-1β, and IL-6. These events collectively lead to microcirculatory disturbance, oxidative stress, and mitochondrial dysfunction, further amplifying hepatic injury and functional decline [10–13].

Among these processes, oxidative stress and mitochondrial impairment are now recognized as central drivers of hepatocyte death across multiple forms of ALI [14, 15]. Reactive oxygen species (ROS) generated from disrupted mitochondrial respiration induce lipid peroxidation, DNA damage, and ATP depletion, thereby sensitizing hepatocytes to apoptotic and necrotic death [16–18]. Persistent ROS accumulation not only aggravates cellular injury but also impairs antioxidant defenses and metabolic homeostasis, establishing a vicious cycle of oxidative damage. In severe cases, this imbalance affects hepatic stellate cells in particular, contributing to their activation and subsequent hepatic fibrogenesis [19, 20]. Despite advances in understanding these molecular mechanisms, effective pharmacological interventions for ALI remain lacking. Current management is largely supportive, emphasizing hemodynamic stabilization, withdrawal of offending agents, and liver transplantation in advanced cases [21–24]. Overall, the therapeutic landscape for ALI remains narrow, with a paucity of effective, diverse, and broadly applicable pharmacological options [25, 26]. The absence of targeted therapeutics capable of limiting early oxidative stress and mitochondrial damage underscores the urgent need for protective approaches that help maintain redox balance and preserve hepatic function during toxic insult.

Nanozymes, nanomaterials that mimic biological enzymes, possess high stability, low cost, tunable catalytic properties, and good operational flexibility, and have been widely used in biological sensing, environmental governance, and biomedicine [27–32]. The antioxidant catalytic activities, such as catalase (CAT)-like, glutathione peroxidase (GPX)-like, and superoxide dismutase (SOD)-like activities, make nanozymes promising candidates for the treatment of ALI. Ceria nanozymes have been utilized to treat liver injury based on their free radical capture capability and anti-inflammatory effects [33, 34]. However, the biosafety of ceria nanozymes needs to be further studied [35]. Prussian blue nanozymes [36, 37] and manganese Prussian blue nanozymes [38] have been demonstrated to treat drug-induced liver injury through scavenging ROS, alleviating oxidative stress, inhibiting hepatocyte apoptosis, and mediating the responses of multi-biological signaling pathways. Moreover, PdPt@MnO_2_ nanozymes [39] with peroxidase, CAT and SOD-like activities showed therapeutic effect for APAP-induced liver injury by scavenging ROS, decreasing inflammatory cytokines, inhibiting recruitment and activation of monocyte/macrophage cells and neutrophils. However, these nanozymes show lower catalytic activity compared with natural enzymes, and the larger particle size and smaller specific surface area severely limit the further improvement of catalytic activity and thus restrict the protective/therapeutic effect. In addition, these nanozymes often lack in vivo self-tracing capability, making it difficult to conveniently observe their metabolic processes in vitro or in vivo. Therefore, the development of nanozymes with higher antioxidant activity, self-tracing ability and good biocompatibility is expected to achieve more efficient protective/therapeutic effects on ALI.

Carbon dots (CDs) are a class of fluorescent nanomaterials with a particle size of less than 10 nm. In recent years, CDs have been proven to possess excellent biosafety and antioxidant catalytic activity that could be applied in the treatment of oxidative stress-related diseases, such as acute lung injury [40], cerebral ischemia-reperfusion injury [41], colitis [42], diabetic skin wound repair [43], and atopic dermatitis [44]. We previously prepared CDs with an SOD-like activity exceeding 10000 U/mg (comparable to that of natural SOD) using the wet oxidation method [41]. Unfortunately, such CD nanozymes have poor fluorescence, making it difficult to achieve good bioimaging. To solve this problem, we developed nitrogen-doped CD nanozymes with a high fluorescence quantum yield (QY) of up to 14%, which can be effectively used for bioimaging to obtain the distribution information of nanozymes in vitro and in vivo [40]. However, their SOD-like activity was only 4000 U/mg. Although they can effectively treat acute lung injury, a relatively high dose (20 mg/kg) was required. Therefore, the development of novel CD nanozymes with high antioxidant activity and favorable fluorescent properties remains in high demand.

**Scheme 1:**
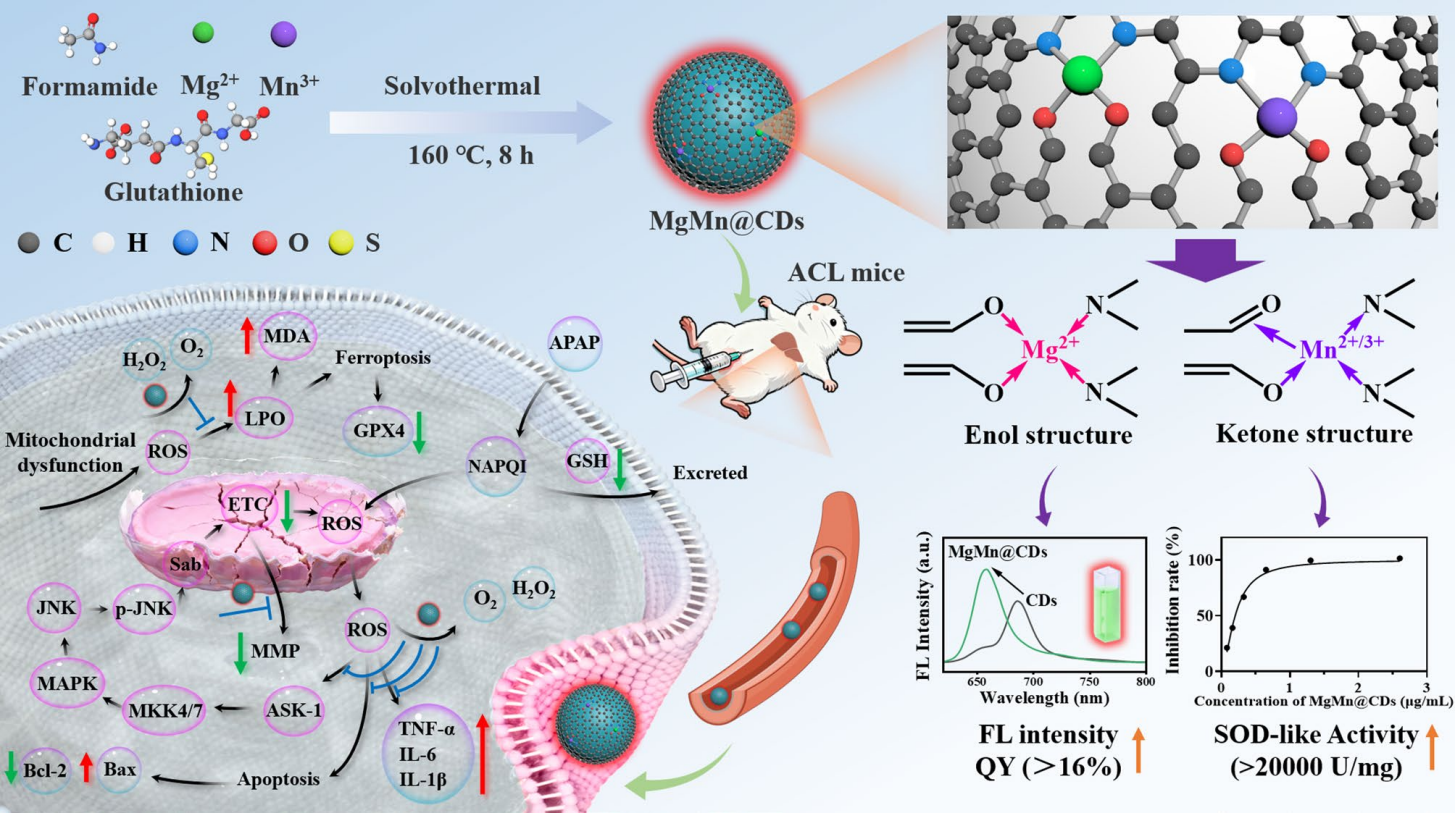
Schematic illustrations of the preparation, properties, and the application of MgMn@CDs in mitigating acetaminophen-induced ALI

Our latest works confirmed that many metal ions can form stable chelates with CDs, thereby effectively regulating their fluorescence and catalytic activity. [45–47] In this work, we synthesized Mg, Mn co-doped CD (MgMn@CD) nanozymes, which possess ultra-high SOD-like activity (> 20000 U/mg) and favorable fluorescent properties (QY > 16%). Structural analysis results show that both Mg and Mn are distributed in the CD structure in a single-atom state. Mg prefers to coordinate with hydroxyl groups, thus increasing the content of enol structure in the CDs by promoting the conversion of ketone structures to enol structures, thereby increasing the rigidity of the CD structure and improving light absorption capacity and fluorescence QY. Mn, on the other hand, synergistically enhances SOD-like activity through coordination with ketone structures and nitrogen-containing structures in the CD. Notably, when the doping amount is moderate, there is less coordination competition between Mg and Mn in the CD structure. Hence, the MgMn@CD nanozymes with both satisfactory fluorescence and high SOD-like activity could be obtained (Scheme 1). Importantly, in vitro experiments confirmed the antioxidant and cytoprotective effects of MgMn@CDs nanozymes. In an APAP-induced hepatotoxicity mouse model, MgMn@CDs nanozymes exerted potent hepatoprotective effects by efficiently scavenging ROS, restoring mitochondrial function, and suppressing apoptosis, ferroptosis, and inflammation. With excellent biocompatibility, favorable hepatic accumulation at the injury site, and coordinated regulation of oxidative stress and apoptotic pathways, MgMn@CDs represent a promising nanozyme platform for mitigating acute liver injury.

## Results and discussion

**Fig. 1:**
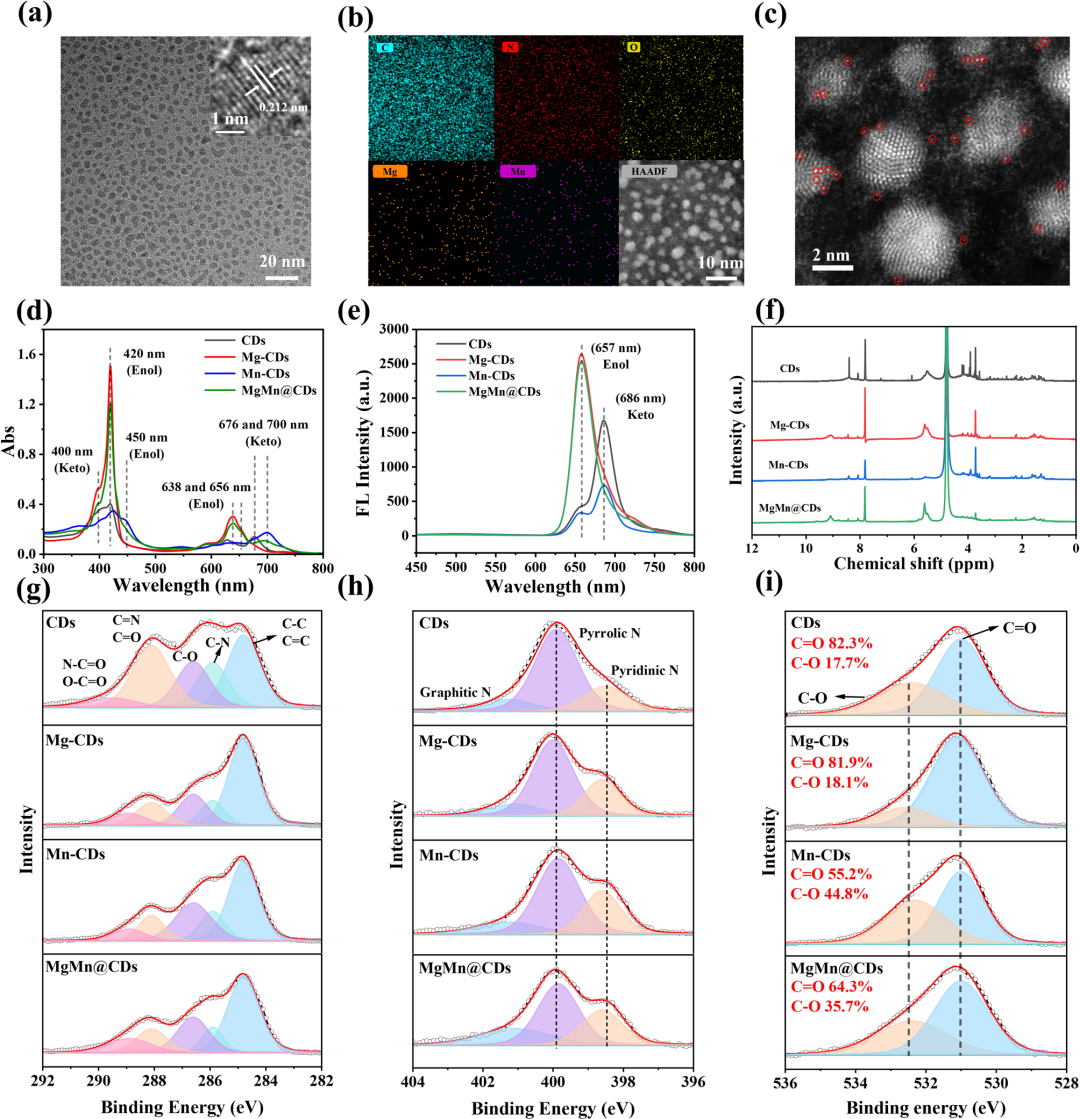
Structural characterization of MgMn@CDs. (**a**) TEM image of MgMn@CDs (inset: high-resolution TEM image). (**b**) Element mapping of C, N, O, Mg, and Mn in MgMn@CDs. (**c**) HAADF-STEM image of MgMn@CDs. UV-Vis absorption spectra (d) fluorescence emission spectra (**e**), and ^1^H NMR (**f**) of CDs, Mg-CDs, Mn-CDs, and MgMn@CDs. C 1 s (**g**), N 1 s (**h**), and O 1 s (**i**) high-resolution XPS spectra of CDs, Mg-CDs, Mn-CDs, and MgMn@CDs

Mg, Mn dual doped CDs (MgMn@CDs) were synthesized via a one-step solvothermal method, using magnesium chloride hexahydrate, manganese acetylacetonate, reduced glutathione, and formamide as precursors, based on our previous work [40, 44, 46, 47]. The morphology of MgMn@CDs was characterized using the transmission electron microscopy (TEM). As shown in Fig. 1a, MgMn@CDs exhibited a uniform distribution, with an average particle size of 2.6 ± 0.4 nm (Fig. S1). The lattice spacing of MgMn@CDs was 0.212 nm, corresponding to the (100) crystal plane of graphite [41]. The energy-dispersive X-ray spectroscopy (EDS) mapping showed that C, N, O, Mg, and Mn elements were uniformly distributed in MgMn@CDs (Fig. 1b), and the high-angle annular dark field scanning TEM (HAADF-STEM) image further demonstrated the single-atom distribution of Mg and Mn in MgMn@CDs (Fig. 1c). Additionally, the metal content was quantified via inductively coupled plasma optical emission spectrometry (ICP-OES). As shown in Table S1, the Mg content in Mg-CDs was 2.66 wt %, while the Mn content in Mn-CDs was 2.40 wt %. For MgMn@CDs, the contents of Mg and Mn were 2.09 wt % and 1.86 wt%, respectively. These results confirm the successful doping of Mg and Mn into CDs, as well as a relatively low coordination competition between the two metal elements.

The optical properties of MgMn@CDs were investigated. Figures 1d and S2 shows the UV-vis absorption spectra of CDs, Mg-CDs, Mn-CDs, and MgMn@CDs. As for CDs without metal doping, there were several obvious absorption peaks in the ranges of 400–430 and 600–700 nm, which correspond to the π − π* and n − π* transitions of conjugated structures containing C = O, C = N, C-O, and C-N [48]. Our previous work [45, 49] demonstrated that the UV-vis absorption peaks of CDs at ~420, 638 and 656 nm related to the enol structure in CDs, while the absorption peaks at 400 and 676 nm were associated with the keto structure in CDs. Meanwhile, the peak at 450 nm of Mn-CDs can be attributed to the absorption of the enol structure, whereas the 700 nm absorption peak was assigned to the keto structure, and the absorption peaks at 420, 638 and 656 nm in Mg-CDs were attributed to the enol structure [44]. Compared with CDs, the absorption peak at ~420, 638 and 656 nm of Mg-CDs significantly increased (Figs. 1d and S2), indicating that doping with Mg^2+^ could promote the transformation of the keto structure to the enol structure in Mg-CDs. Therefore, it was inferred that Mg^2+^ might possess a higher affinity for the enol structure. For Mn-CDs, the absorbance of the enol-related absorption peaks at ~420, 638 and 656 nm decreased while that of at 700 nm related to keto increased (Fig. 1d). These results suggested that Mn doping promoted the transformation from enol to keto structure in CDs, and Mn ions exhibited a higher coordination affinity toward the double-bonded oxygen atoms in the keto structure. For MgMn@CDs, the characteristic absorption peaks related to Mg doped structure appeared at ~420 nm and ~638 nm, while those related to Mn doped structure was detected at 700 nm. Notably, the intensities of the characteristic peaks of MgMn@CDs at ~420 and ~638 nm were lower than those of Mg-CDs. while that at 700 nm of MgMn@CDs was lower than that of Mn-CDs. These results also confirmed the successful co-doping of Mg and Mn in MgMn@CDs. The fluorescence emission spectra of CDs, Mg-CDs, Mn-CDs, and MgMn@CDs are shown in Fig. 1e. Under the excitation wavelength of 420 nm, the fluorescence emission spectrum of CDs exhibits a maximal emission peak at 686 nm and a shoulder peak at 657 nm. In our previous work [45], the emission peaks at 657 and 686 nm were revealed to correspond to the enol and keto structure, respectively. Mg-CDs exhibited an optimal emission peak at 657 nm with an enhanced intensity, while the emission peak at 686 nm disappeared, indicating that Mg^2+^ prefers to coordinate with enol in CDs, and therefore facilitating keto-to-enol transformation. The fluorescence emission spectrum and intensity of MgMn@CDs were similar to those of Mg-CDs, indicating that the coordination structure of Mg^2+^ in CD skeleton serves as the dominant fluorescent emission site in MgMn@CDs while the coordination of Mn affected the fluorescence less. The average fluorescence lifetime of MgMn@CDs was determined to be 3.14 ns (Fig. S3 and Table S2). Additionally, MgMn@CDs exhibit a high absolute fluorescence QY of 16.4% (Table S2). The high QY and long emission wavelength of MgMn@CDs make them suitable for bioimaging, especially in vivo bioimaging.

The proton magnetic resonance (^1^H-NMR) spectra of CDs, Mg-CDs, Mn-CDs, and MgMn@CDs are shown in Fig. 1f. As for CDs without metal doping, the peaks in the range of 3.0–4.3.0.3 ppm were attributed to the H in O-CH_x_. The peak near 5.5 ppm was attributed to the H of alkenes, and the peak near 7.8 ppm was attributed to the H of aromatic rings. Compared to the [1]H NMR spectrum of CDs, the peak at approximately 5.5 ppm enhanced in Mg-CDs but weakened in Mn-CDs, which indicates that the doping Mg (II) preferred to coordinate with the enol form, while Mn preferred to coordinate with the keto form structure in CDs. The peak intensity near 5.5 ppm in MgMn@CDs is lower than that in Mg-CDs, which is attributed to the coordination of Mn. The Fourier transform infrared (FT-IR) spectra of CDs, Mg-CDs, Mn-CDs, and MgMn@CDs are shown in Figure S4. In the FT-IR spectrum of CDs, the peaks at 3420 and 3140 cm^− 1^ were corresponded to the stretching vibrations of O-H and N-H, respectively. The peaks at 1680 and 1615 cm^− 1^ were attributed to the stretching vibrations of C = O (ketone, carboxyl, or amide groups) and C = C/C = N, respectively [42]. In Mg-CDs, Mn-CDs, and MgMn@CDs, the intensity of the stretching vibration band corresponding to the N-H bond decreased and the peak at 1680 cm^− 1^ shifted to 1670 cm^− 1^. Meanwhile, the intensities of the bands at 1552 cm⁻¹ (attributed to the stretching vibration of the aromatic ring skeleton or amide II band) and 1308 cm⁻¹ (attributed to the stretching vibration of the amide III band) increased. This phenomenon indicated that the metal (Mg and Mn) ions coordinate with the amide bonds of CDs.

The X-ray photoelectron spectroscopy (XPS) was utilized for characterizing the elemental composition and valence states of CDs and MgMn@CDs. As shown in Figure S5, CDs, Mg-CDs, Mn-CDs and MgMn@CDs exhibited four peaks at 164, 285, 400, and 532 eV, which correspond to the characteristic peaks of S 2p, C 1 s, N 1 s, and O 1 s, respectively. In addition, after Mn or Mg was doped into the CDs, the characteristic peaks attributed to Mn 2p and Mg 1 s were observed at 642 eV and 1304 eV, respectively [44]. Mn 2p high resolution (HR)-XPS spectra of Mn-CDs and MgMn@CDs exhibited two peaks at binding energies of ~640.5 and ~652.3 eV (Figure S6), corresponding to Mn 2p_3/2_ and Mn 2p_1/2_, respectively. The Mn 2p_3/2_ peak could be fitted with two peaks at 640.4 and 642.3 eV, corresponded to Mn^2+^ and Mn^3+^, respectively. The change in the valence state of some Mn ions from +3 to +2 could be attributed to the reduction of Mn^3+^ during synthesis, a process involving a high concentration of glutathione that can reduce Mn ions in higher oxidation states. The coexistence of Mn^3+^ and Mn^2+^ might have enabled the disproportionation of O_2_^•^⁻ to possess multiple initiation pathways, thereby enhancing the SOD-like enzyme activity. In Mg 1 s HR-XPS spectra of Mg-CDs and MgMn@CDs (Figure S7), the peak at 1303.7 eV corresponded to Mg^2+^. The HR-C 1 s spectra of Mg/Mn-CDs is shown in Fig. 1g. There were five peaks at 284.8, 285.9, 286.5, 288.1, and 289.1 eV, corresponding to C = C/C-C, C-N, C-O, C = N/C = O, and O-C = O/N-C = O bonds, respectively. After Mn/Mg doping, the carbon content with the medium and highest binding energy (C-N, C-O, C = N/C = O, and O-C = O/N-C = O) decreased. Combined with the analysis of ^1^H-NMR, it was speculated that the loading of Mn/Mg caused significant changes in the structure of the CDs. Furthermore, after Mg doping, the carbon with the lowest binding energy content (C = C/C-C) increased, which further confirmed that Mg doping leading to the conversion of the ketone to the enol structure in the CDs. In the N 1 s HR-XPS spectrum of CDs (Fig. 1h), there were three peaks at 401.2, 399.9, and 398.5 eV, which corresponded to graphitic N, pyrrolic N, and pyridinic N, respectively. Compared with CDs, the binding energy of pyridinic N of Mn-CDs increased by 0.1 eV, while that of pyrrolic N of Mn-CDs decreased by 0.1 eV. This change was attributed to the partial electron transfer from Mn ions to pyrrolic N, which formed a backdonation π-bond and resulted in an increased electron cloud density around pyrrolic N. In contrast, partial electrons from pyridinic N in Mn-CDs were transferred to Mn ions to form a σ bond, leading to a reduced electron cloud density around pyridinic N. Meanwhile, compared with CDs, the binding energies of both pyridinic N and pyrrolic N in Mg-CDs were elevated. This was due to the stable valence state of Mg^2+^, which accepted electrons from pyridinic N and pyrrolic N to form σ bonds during coordination. Furthermore, the variation in binding energy of MgMn@CDs became more similar to that of Mn-CDs. This was presumably because Mn ions exhibited higher coordination affinity for pyrrolic N and pyridinic N compared with Mg^2+^. There were two peaks at 531 and 532.5 eV in O 1 s HR-XPS spectrum of CDs (Fig. 1i), corresponding to C = O and C-O, respectively. Compared with CDs, the content of O with high binding energy (corresponding to C-O bonds) in Mn-CDs increased significantly (from 17.7% to 44.8%), while the content of O with low binding energy (corresponding to C = O bonds) decreased obviously (from 82.3% to 55.2%). This result indicated that Mn ions coordinated with the O-containing functional groups on the surface of CDs. In contrast, the binding energies of C-O and C = O in Mg-CDs were increased. This demonstrated that Mg^2+^ coordinated with the O-containing functional groups of CDs via the formation of σ bonds. In MgMn@CDs, there was no significant change in the binding energies of C-O and C = O, while the content of C-O increased. This was attributed to the coordination of Mg and Mn ions with the O-containing functional groups in CDs, which collectively led to this result. These results indicated that during the synthesis of CDs, Mg and Mn ions coordinated with the precursors, leading to significant changes in the structure and functional group composition of CDs. These changes were closely related to the enhancement of fluorescence QY by Mg^2+^ and the improvement of SOD-like activity of CDs by Mn ions.

**Fig. 2:**
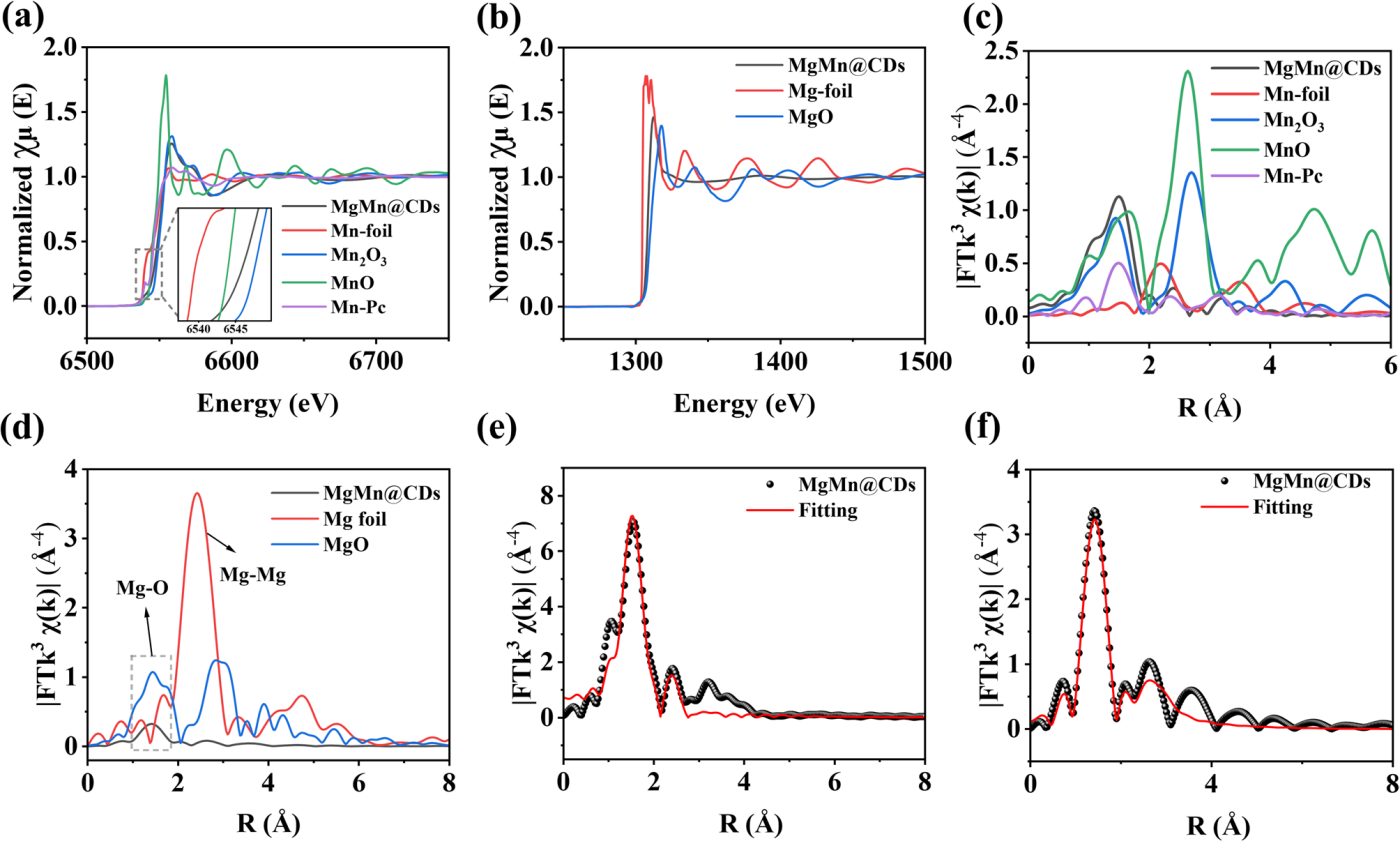
Structural characterization by XANES and EXAFS. (**a**) Mn and (**b**) Mg K-edge XANES spectra of MgMn@CDs and reference samples. Fourier transforms of (**c**) Mn and (**d**) Mg K-edge EXAFS spectra of MgMn@CDs and reference samples. (**e**) Mn and (**f**) Mg K-edge EXAFS (points) and fitting curve (line) for MgMn@CDs

The valence states and coordination environments of Mg and Mn in MgMn@CDs were investigated by X-ray absorption spectroscopy. A variety of Mn reference compounds (including Mn-phthalocyanine (PC) with a well-defined MnN_4_ structure, Mn foil, and Mn oxides) and Mg reference compounds (Mg foil and MgO) were included to compare with MgMn@CDs. In the Mn K-edge X-ray absorption near-edge structure (XANES) spectrum (Fig. 2a), the absorption edge of MgMn@CDs was located between those of MnO and Mn_2_O_3_, and was equally close to both. This result suggested that the valence state of Mn in MgMn@CDs was +2 ~ 3, with a similar proportion of Mn^2+^ to Mn^3+^. In the Mg K-edge XANES spectrum (Fig. 2b), the absorption edge of MgMn@CDs was close to that of MgO. This result implied that Mg in MgMn@CDs mainly existed in the +2 valence state. These findings were consistent with XPS analysis (Figures. S6 and S7). The Mn K-edge Fourier-transformed extended X-ray absorption fine structure (FT-EXAFS) spectra of MgMn@CDs and reference samples were presented in Fig. 2c. The main peak of MgMn@CDs was located at 1.5 Å, which similar with the characteristic peaks of both Mn-Pc and Mn oxides. Furthermore, Mn-Mn bonding at 2.2 Å was not observed. In Mg K-edge FT-EXAFS spectra of MgMn@CDs and reference samples (Fig. 2d), the main peak of MgMn@CDs was located at 1.4 Å, corresponding to the Mg-N/O coordination in the first coordination shell. Besides, Mg-Mg bonding was not observed at 2.3 Å. The wavelet transform (WT) analysis of the Mn K-edge EXAFS of MgMn@CDs suggested that, compared with the spectra of Mn foil, MnO, and Mn_2_O_3_, the WT of MgMn@CDs exhibited a major peak centered at 4.5 Å^−1^ (Figure S8), which was attributed to Mn-N/O bonding. However, the absence of the signal corresponding to Mn-Mn (~7.8 Å^−1^) suggested that aggregated Mn species were absent. In the WT of Mg K-edge EXAFS, the WT contour plot of MgMn@CDs exhibits an intensity peak around 5 Å^−1^, distinguished from that of Mg foil (Figure S9). These results indicated that both Mn and Mg existed in MgMn@CDs in the form of coordination with N/O, and Mg and Mn were dispersed in MgMn@CDs as a single atom. In order to better clarify the coordination environments and structure around the single-atom Mg and Mn centers, least-squares fitting of FT-EXAFS spectra of Mg and Mn K-edge was performed. The FT-EXAFS fitting plots for different scattering paths were shown in Figure S10, with the refined parameters listed in Tables S3 and S4. The best-fit results clearly indicated that the Mn-N/O coordination number of the first layer of Mn atoms in MgMn@CDs was approximately 3.2 (Fig. 2e and Table S3), while that of Mg atoms (Mg-N/O coordination number) was about 3.6 (Fig. 2f and Table S4). Therefore, it can be speculated that both Mn and Mg existed in MgMn@CDs in a single-atomic form through coordination with 3–4 N/O atoms.

**Fig. 3:**
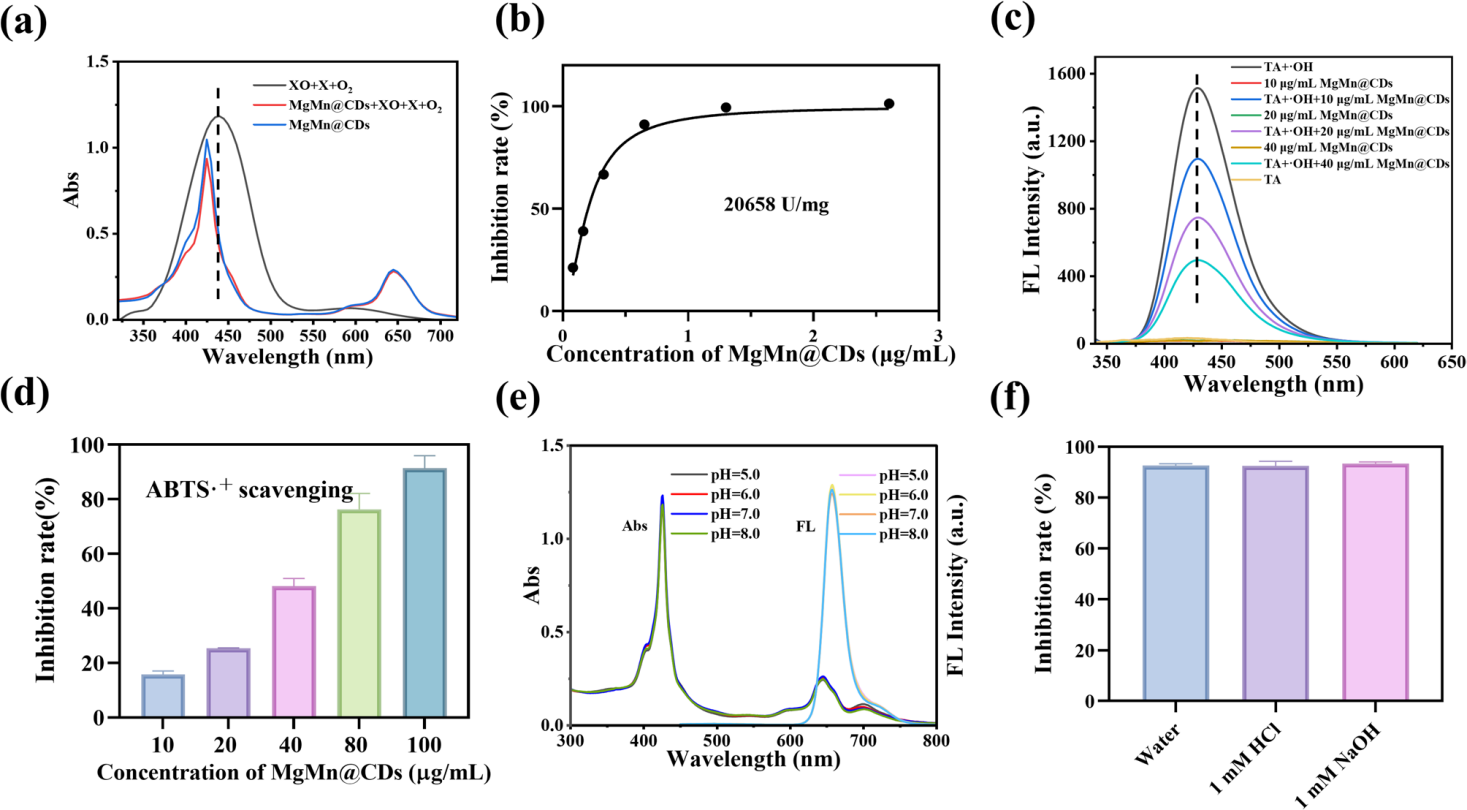
Antioxidant properties and stability of MgMn@CDs. (**a**) O_2_^•−^ scavenging activity of MgMn@CDs (final concentration of 0.65 µg/mL, X: xanthine, XO: xanthine oxidase). (**b**) SOD-like activity of MgMn@CDs. (**c**) •OH and (**d**) ABTS^•+^ scavenging activity of MgMn@CDs. (**e**) Stability of optical properties of MgMn@CDs under different pH values. (**f**) SOD-like activity of MgMn@CDs (final concentration of 0.65 μg/mL) after pretreatment in 1 mM HCl and 1 mM NaOH solutions for 1 h

The free radical scavenging capacity of MgMn@CDs was tested. O_2_^•−^ was generated by the reaction of xanthine and O_2_ catalyzed by xanthine oxidase. O_2_^•−^ can reduce the WST-1 to form formazan dye with strong absorption at 450 nm. SOD mimics can inhibit this chromogenic reaction. As shown in Fig. 3a, the addition of MgMn@CDs significantly reduced the intensity of the characteristic absorption peak around 450 nm, indicating that MgMn@CDs possess excellent SOD-like activity and can catalyze the dismutation reaction of O_2_^•−^. The SOD-like activity of MgMn@CDs was quantitative determined to be 20,658 U/mg via the WST-1 method (Fig. 3b). MgMn@CDs exhibited superior catalytic performance and satisfactory fluorescence properties, and these properties were far better than those of many previously reported CD-based SOD nanozymes. (Table S5). Hydroxyl radicals (·OH) with the highest reduction potential of 2.31 V are the most toxic ROS in organisms. Therefore, ·OH scavenging capacity of MgMn@CDs was also determined. The generation of ⋅OH was achieved by irradiating H₂O₂ under ultraviolet light. Terephthalic acid (TA) was used as a probe and reacts with ⋅OH to form 2-hydroxyterephthalic acid, which exhibits a characteristic fluorescence peak at approximately 430 nm. As shown in Fig. 3c, the fluorescence emission intensity around 430 nm gradually decreased with the increase in the concentration of MgMn@CDs, demonstrating the·OH scavenging capacity of MgMn@CDs. In addition, as shown in Fig. 3d, MgMn@CDs also exhibited a strong scavenging ability for ABTS·⁺, and when its concentration reached 100 µg/mL, the scavenging rate for ABTS·⁺ was as high as 90%. To evaluate the practical application potential of MgMn@CDs, their stability under various conditions was systematically investigated. As shown in Fig. 3e, the fluorescence emission and UV-vis absorption spectra of MgMn@CDs remained stable within the pH range of 5.0–8.0, indicating that the fluorescence emission structures of MgMn@CDs remained stable under most physiological conditions, providing a guarantee for both in vitro and in vivo imaging. After treated with extreme environments of 1 mM HCl and 1 mM NaOH (Fig. 3f), the SOD-like activity of MgMn@CDs did not decrease significantly, which was not observed in nature enzymes, suggesting that their catalytic active center possesses outstanding stability. Furthermore, as shown in Figure S11, after 7 days of storage in water, PBS, and FBS, MgMn@CDs still maintained stability in terms of both optical properties and SOD-like activity, thereby demonstrating favorable long-term storage performance. Meanwhile, within the temperature range of 25–45 °C, the SOD-like activity of MgMn@CDs did not change, thus showing good thermal adaptability. Therefore, MgMn@CDs combine high SOD-like activity, strong fluorescence, wide pH tolerance, long-term storage stability, and thermal stability, and thus exhibit promising potential in complex scenarios such as biosensing and disease treatment.

### MgMn@CDs scavenge ROS in vitro

**Fig. 4:**
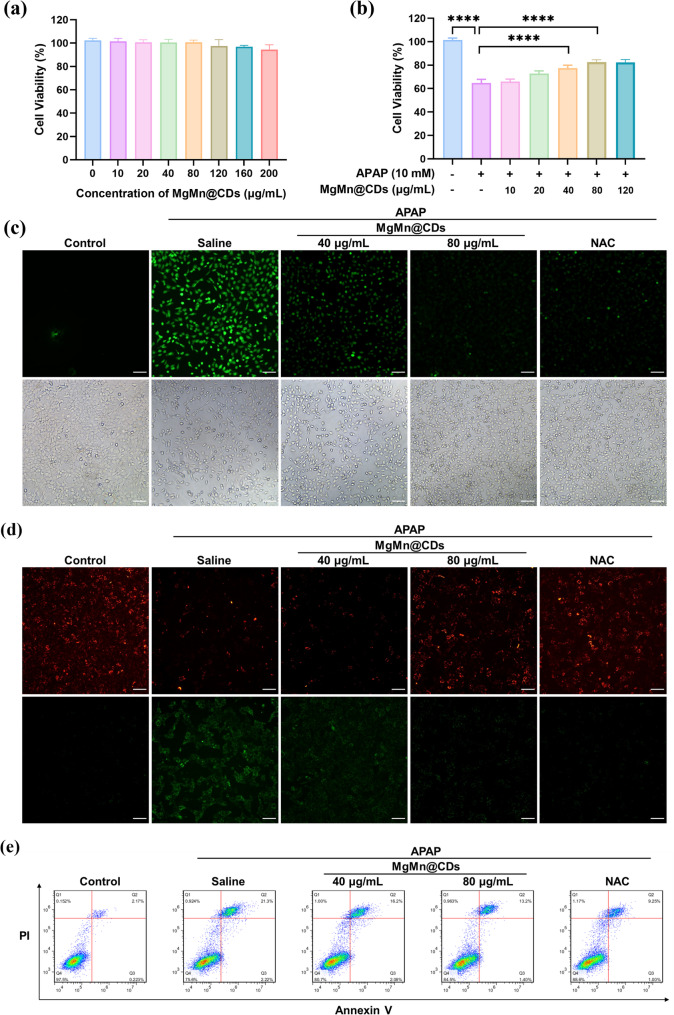
MgMn@CDs protected APAP-induced hepatocyte apoptosis by resisting oxidative stress in vitro. (**a**) Cytotoxic effects of MgMn@CDs on AML12 cells (*n* = 3); (**b**) Viabilities of AML12 cells treated with APAP and different doses of MgMn@CDs (*n* = 3); (**c**) ROS detection by DCFH-DA staining in AML12 cells pretreated with different doses of MgMn@CDs followed by APAP, scale bar = 100 μm; (**d**) JC-1 staining of AML12 cells pretreated with different doses of MgMn@CDs followed by APAP, scale bar = 100 μm; (**e**) Annexin V-FITC/PI staining of AML12 cells pretreated with different doses of MgMn@CDs followed by APAP. All data are presented as mean *±* SD from three independent experiments. *p < 0.05, **p < 0.01, ***p < 0.001, ****p < 0.0001

We investigated the cytotoxicity and potential antioxidant activity of MgMn@CD nanozymes in vitro using AML-12 cells, a well-established cellular model for investigating hepatic oxidative stress and detoxification mechanisms [50]. The cytocompatibility of MgMn@CDs was first evaluated using the MTT assay. As depicted in Fig. 4a, MgMn@CDs exhibited negligible cytotoxicity across a wide concentration range (up to 200 µg/mL), confirming their excellent cellular compatibility and suitability for biological applications. Oxidative stress plays a pivotal role in the pathogenesis of APAP-induced liver injury (AILI), where excessive generation of ROS disrupts mitochondrial respiration, weakens antioxidant defenses, and ultimately leads to hepatocyte injury [51]. Accordingly, APAP-induced oxidative stress provides a well-established experimental model for evaluating the antioxidant potential and hepatoprotective mechanisms of candidate nanozymes. We next employed this model to examine the protective effects of MgMn@CDs against APAP-induced oxidative injury. Exposure to 10 mM APAP decreased cell viability to approximately 60%, indicating substantial oxidative damage. Remarkably, treatment with MgMn@CDs significantly enhanced cell viability in a concentration-dependent manner. Pronounced protective effects were observed at 40 µg/mL, with a plateau reached at 80 µg/mL (Fig. 4b). These concentrations were therefore selected for subsequent mechanistic investigations. This dose-dependent protection highlights the bioactive yet biocompatible nature of the nanozymes, suggesting that MgMn@CDs effectively modulate intracellular redox balance without eliciting cytotoxic effects.

Excessive ROS accumulation is widely recognized as a key pathogenic driver in AILI, causing mitochondrial depolarization and cellular damage [52]. To verify the antioxidant mechanism of MgMn@CDs, intracellular ROS levels were visualized using the DCFH-DA fluorescent probe. As shown in Fig. 4c, cells in the APAP+saline group displayed intense green fluorescence, indicative of substantial ROS accumulation. In contrast, pretreatment with MgMn@CDs or the positive control NAC markedly reduced fluorescence intensity, demonstrating efficient ROS scavenging capacity. These findings indicate that MgMn@CD nanozymes act as potent ROS scavengers, effectively alleviating intracellular oxidative stress.

To further characterize the antioxidant defense response, oxidative stress markers including malondialdehyde (MDA), SOD, and glutathione (GSH) were quantified. MDA, a byproduct of lipid peroxidation, reflects the extent of oxidative membrane damage, while SOD and GSH serve as key endogenous antioxidants that maintain redox equilibrium [53]. The results showed that MDA levels were markedly elevated in the APAP+saline group compared with the control, reflecting severe lipid peroxidation and oxidative injury. Treatment with MgMn@CDs or NAC significantly reduced MDA content, with MgMn@CDs exhibiting a restorative effect comparable to NAC. Conversely, the activities of SOD and the intracellular GSH content were significantly decreased in the APAP+saline group, indicating impaired antioxidant capacity. Both MgMn@CDs and NAC treatments effectively restored SOD activity and GSH levels, with the MgMn@CDs-treated group showing a pronounced recovery relative to the APAP+saline group (Figure S12).

Finally, the mRNA levels of key proinflammatory cytokines, including TNF-α, IL-1β, and IL-6, were determined by RT-qPCR to assess the anti-inflammatory effects of MgMn@CDs. As shown in Figure S13, APAP administration led to a marked upregulation of all three cytokines compared with the control group, reflecting a strong hepatic inflammatory response. Notably, cells pretreated with 80 µg/mL MgMn@CDs exhibited mRNA levels of TNF-α, IL-1β, and IL-6 comparable to those observed in the NAC-treated group, suggesting that MgMn@CDs effectively mitigate APAP-induced inflammatory responses by suppressing the expression of proinflammatory mediators.

Collectively, these results confirm that MgMn@CDs effectively attenuate APAP-induced oxidative damage by suppressing ROS accumulation, restoring the intrinsic antioxidant network, and alleviating the inflammatory response, thereby providing a robust in vitro foundation for their subsequent in vivo hepatoprotective evaluation.

### MgMn@CDs restore mitochondrial function and alleviate apoptosis

Mitochondria serve as the primary energy centers of hepatocytes and are particularly vulnerable to oxidative damage during APAP overdose. The reactive metabolite *N*-acetyl-*p*-benzoquinone imine (NAPQI) generated during APAP metabolism disrupts mitochondrial electron transport, leading to ATP depletion, mitochondrial membrane depolarization, and eventually apoptotic cell death [54]. Therefore, restoring mitochondrial function and maintaining membrane potential are crucial for alleviating APAP-induced hepatocellular injury. Given the potent antioxidant activity of MgMn@CDs demonstrated above, we next examined whether these nanozymes could protect mitochondrial integrity and suppress downstream apoptotic cascades in hepatocytes.

To evaluate mitochondrial membrane potential (MMP), a sensitive indicator of mitochondrial health, JC-1 staining was performed. As shown in Fig. 4d, cells in the APAP+saline group exhibited a marked loss of red fluorescence accompanied by an increase in green fluorescence, reflecting severe depolarization of the mitochondrial membrane. In contrast, cells treated with MgMn@CDs or NAC retained strong red fluorescence and reduced green signal intensity, indicating that mitochondrial polarization was largely preserved. These results demonstrate that MgMn@CDs effectively stabilize mitochondrial membrane potential, thereby preventing mitochondrial dysfunction triggered by APAP metabolism. Given that mitochondrial impairment often precedes apoptotic progression, the anti-apoptotic effect of MgMn@CDs was further investigated by Annexin V/PI dual staining. The control group exhibited minimal apoptosis (2.17%), consistent with normal cellular viability. In contrast, APAP challenge markedly increased the apoptotic rate to 21.30% in the APAP+saline group, confirming severe cytotoxicity. Remarkably, MgMn@CDs treatment reduced the apoptotic cell population in a dose-dependent manner, with the apoptotic rates decreasing to 16.20% and 13.20% at concentrations of 40 and 80 μg/mL, respectively. Moreover, the anti-apoptotic effect of 80 μg/mL MgMn@CDs was comparable to that of NAC (9.25%) (Fig. 4e). This significant reduction indicates that MgMn@CDs effectively attenuate APAP-induced hepatocyte apoptosis, likely through the preservation of mitochondrial function and suppression of oxidative stress–induced cell death signaling. Mechanistically, these observations suggest that MgMn@CDs intervene upstream of the mitochondrial apoptotic cascade by maintaining redox homeostasis and preventing mitochondrial membrane disruption, thereby limiting cytochrome *c* release and caspase activation. Previous studies have established that oxidative stress-driven mitochondrial permeability transition is a critical initiating event in APAP-induced apoptosis [55, 56]; thus, by preserving mitochondrial polarization and reducing ROS burden, MgMn@CDs interrupt this pathological sequence at an early stage. The observed dose-dependent efficacy further implies that the catalytic centers of MgMn@CDs may facilitate sustained redox cycling and protect mitochondrial enzymes from NAPQI-induced inactivation.

In summary, MgMn@CD nanozymes confer comprehensive cytoprotective effects in hepatocytes by simultaneously stabilizing mitochondrial membrane potential and suppressing apoptosis. These findings, together with their potent ROS-scavenging activity, establish that MgMn@CDs mitigate APAP-induced hepatocellular injury through a dual mechanism involving both oxidative stress suppression and mitochondrial preservation.

### Biosafety and in vivo distribution of MgMn@CDs

**Fig. 5:**
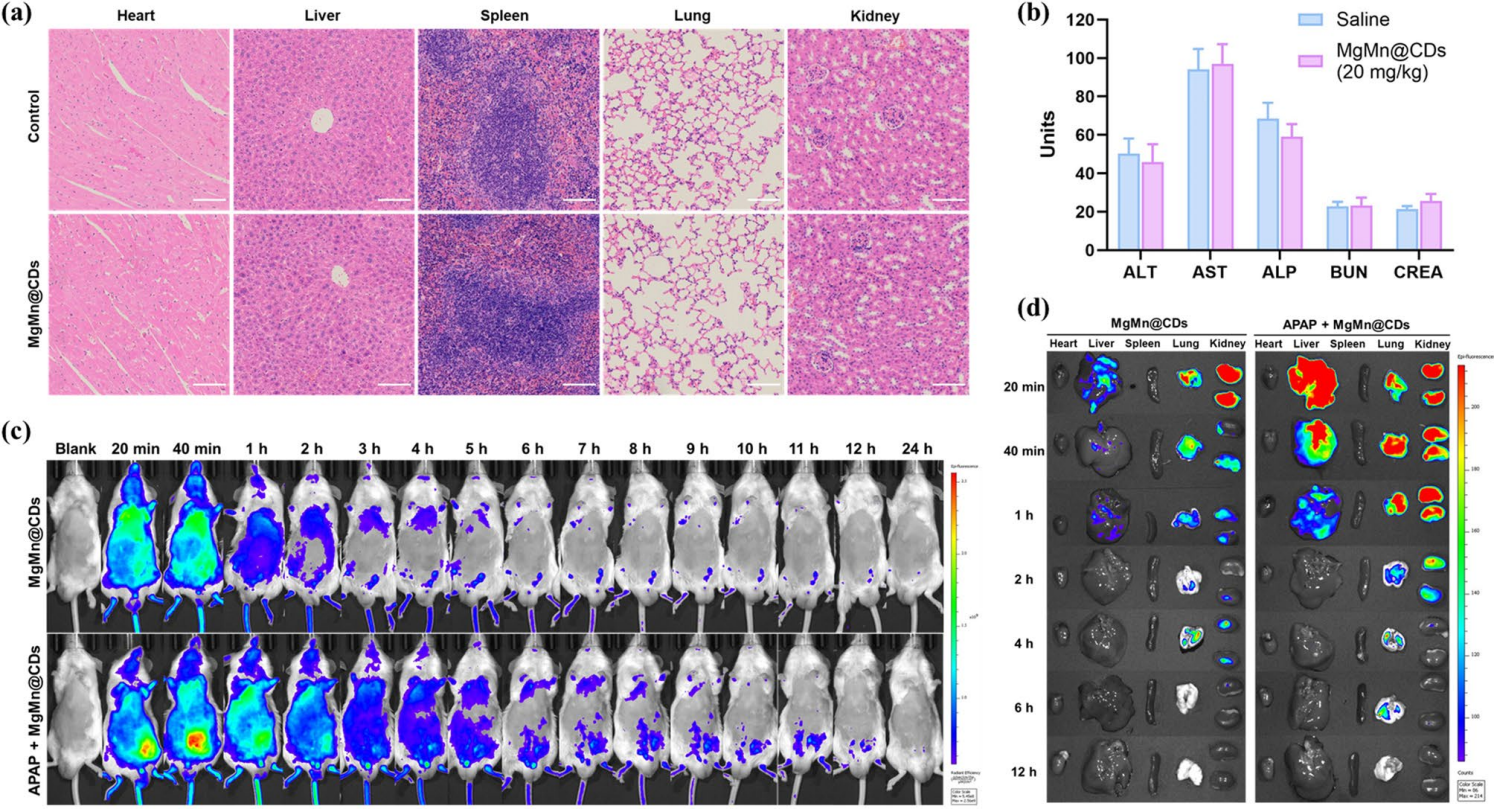
Biological safety evaluation and biodistribution analysis of MgMn@CDs. (**a**) H&E staining of major organs (heart, liver, spleen, lung, and kidney) for in vivo toxicity evaluation of 30 days after intraperitoneal administration of MgMn@CDs to (*n* = 6, scale bar = 100 μm); (**b**) Serum levels of hepatic and renal function markers after intraperitoneal injection of MgMn@CDs (*n* = 6); (**c**) In vivo fluorescence imaging of MgMn@CDs biodistribution (*n* = 6); (**d**) Ex vivo fluorescence imaging of MgMn@CDs accumulation in major organs (from left to right: heart, liver, spleen, lung, and kidney) (*n* = 6). All data are presented as mean *±* SD from three independent experiments

Before evaluating the hepatoprotective efficacy of MgMn@CDs nanozymes in the AILI model, it was essential to assess their biosafety and biodistribution characteristics to ensure suitability for in vivo applications. Initially, mice treated with MgMn@CDs for 30 days were sacrificed, and their heart, liver, spleen, lungs, and kidneys were harvested for hematoxylin-eosin (H&E) staining to assess systemic safety. As shown in Fig. 5a, H&E staining revealed intact tissue architecture across all examined organs, with no evidence of necrosis, inflammatory infiltration, or hemorrhagic lesions compared with the saline-treated control group. This morphological integrity indicates excellent biocompatibility of MgMn@CDs and the absence of overt systemic toxicity during prolonged exposure.

To further validate these findings at the biochemical level, a panel of hepatic and renal function markers, including alanine aminotransferase (ALT), aspartate aminotransferase (AST), alkaline phosphatase (ALP), blood urea nitrogen (BUN), and creatinine (CREA), was measured (Fig. 5b). All parameters remained within the normal physiological range, with no statistically significant differences between MgMn@CDs-treated and control groups. These data confirm that MgMn@CDs exhibit negligible in vivo toxicity and favorable biocompatibility.

Next, the in vivo distribution and hepatic retention of MgMn@CDs were investigated by fluorescence imaging. Mice treated with MgMn@CDs exhibited a strong fluorescence signal throughout the body as early as 20 min post-injection, indicating rapid distribution (Fig. 5c). The hepatic fluorescence intensity gradually declined and was nearly undetectable by 5 h post-injection, indicating effective systemic clearance and favorable metabolic compatibility. This fluorescence decay is consistent with pharmacokinetic analysis, which revealed a plasma half-life (t_1/2_) of 61.15 min, suggesting that MgMn@CDs would be largely cleared from systemic circulation by 7 h (estimated as 7 × t_1/2_, Figure S14). In contrast, the APAP+MgMn@CDs group exhibited significantly stronger and more persistent fluorescence, which lasted up to 12 h before dissipation (Fig. 5c). Ex vivo imaging of major organs further corroborated these observations, with the liver from the APAP+MgMn@CDs group displaying noticeably stronger and more persistent fluorescence compared with the control group (Fig. 5d). Such prolonged hepatic retention suggests that APAP-induced tissue injury may alter local vascular permeability or macrophage uptake dynamics, thereby enhancing the accumulation and retention of MgMn@CDs at the injury site.

Collectively, these findings demonstrate that MgMn@CDs nanozymes possess excellent systemic biosafety, efficient metabolic clearance, and strong hepatic tropism. Their rapid liver accumulation coupled with injury-responsive retention provides an optimal pharmacokinetic foundation for protective intervention in APAP-induced hepatotoxicity.

### MgMn@CDs mitigate APAP-induced liver injury in vivo and regulate apoptosis and ferroptosis

**Fig. 6:**
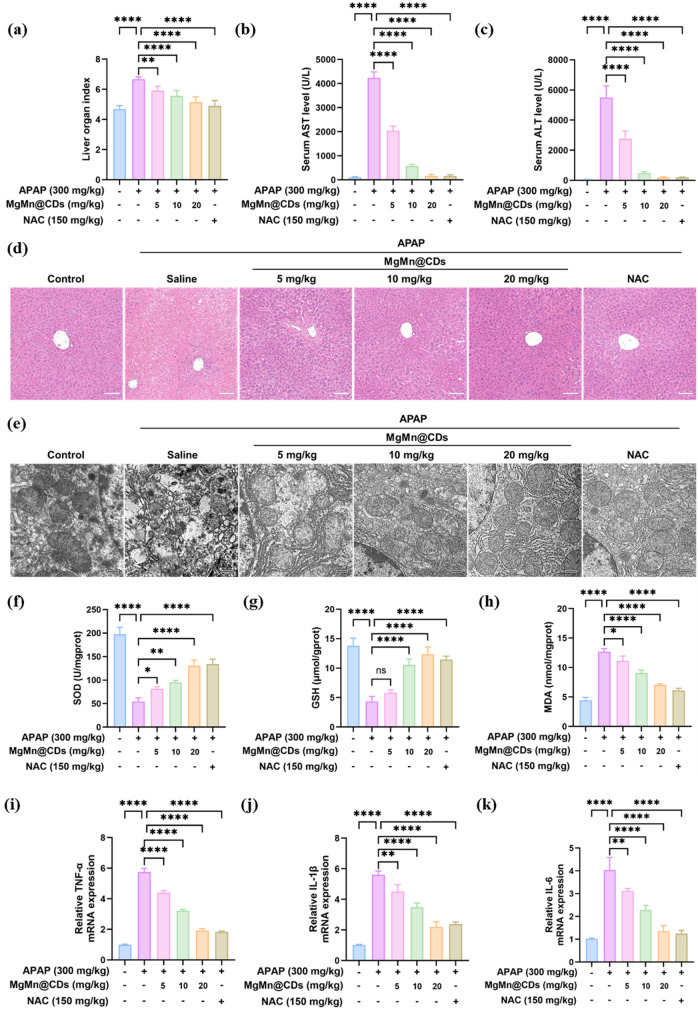
Hepatoprotective effect of MgMn@CDs on AILI. (**a**) Liver organ index in mice pretreated with MgMn@CDs before APAP administration (*n* = 6); Serum levels of AST (**b**) and ALT (**c**) after APAP intoxication (*n* = 6 per group); (**d**) Representative H&E staining images of the liver tissue sections (*n* = 6, scale bars: 100 μm); (**e**) Representative TEM images of mitochondria in liver tissue, scale bars: 500 nm; Levels of oxidative stress biomarkers of SOD (**f**), GSH (**g**) and MDA (**h**); mRNA levels of key proinflammatory cytokines TNF-α (**i**), IL-1β (**j**), and IL-6 (**k**). All data are presented as mean *±* SD from three independent experiments. *p < 0.05, **p < 0.01, ***p < 0.001, ****p < 0.0001

Inspired by the robust protective effects of MgMn@CDs observed in vitro and their favorable liver-targeting profile, we next evaluated their hepatoprotective efficacy in APAP-induced acute liver injury in mice. As mentioned, APAP overdose is known to cause hepatocellular injury, mitochondrial dysfunction, and systemic oxidative stress, ultimately leading to elevated liver enzymes and inflammation. Given this pathophysiological cascade, we investigated whether MgMn@CDs could mitigate these deleterious effects when administered before APAP exposure. First, liver organ indices were measured to assess hepatic swelling. As shown in Fig. 6a, APAP-treated mice displayed a significant increase in liver index, reflecting severe hepatomegaly and tissue edema. Treatment with MgMn@CDs at low (5 mg/kg), medium (10 mg/kg), and high (20 mg/kg) doses progressively reduced liver swelling in a dose-dependent manner, with the high-dose group approaching values comparable to those of NAC-treated mice.

Serum biochemical markers further substantiated these observations. ALT and AST, widely recognized indicators of hepatocellular damage, were markedly elevated in the APAP+saline group, consistent with severe liver injury. Administration of MgMn@CDs resulted in a dose-dependent reduction of both enzymes. Specifically, the 5 mg/kg and 10 mg/kg groups exhibited moderate decreases, whereas the 20 mg/kg group achieved enzyme levels nearly equivalent to those of NAC treatment (Fig. 6b and c). These results demonstrate that MgMn@CDs effectively restore liver function and alleviate hepatocellular injury in a dose-dependent manner. Histopathological analysis provided additional confirmation of hepatoprotective effects at the tissue level. H&E staining revealed extensive necrosis, hepatocyte disarray, and inflammatory infiltration in the APAP+saline group. By contrast, the MgMn@CDs-treated groups exhibited progressively improved liver architecture, with the 20 mg/kg group showing clear and orderly arranged hepatic cords, without obvious expansion or compression of the hepatic sinusoids, and no signs of hemorrhage or deeply stained degeneration (Fig. 6d). These findings indicate that MgMn@CDs not only preserve cellular integrity but also mitigate the inflammatory and necrotic consequences of APAP overdose.

To investigate the underlying cellular mechanisms, transmission electron microscopy (TEM) was performed on hepatocytes isolated from liver tissues. In APAP-treated mice, mitochondria were severely swollen with disrupted cristae and compromised membranes, which are typical features of ferroptosis. MgMn@CDs treatment progressively restored mitochondrial ultrastructure in a dose-dependent manner, with high-dose administration maintaining intact double membranes and well-organized cristae (Fig. 6e). These results demonstrate that MgMn@CDs preserve mitochondrial integrity, a critical factor in maintaining hepatocellular viability under oxidative stress.

Given that APAP-induced liver injury involves excessive apoptosis and ferroptosis, we conducted Western blot analysis to detect the changes in the expression levels of key regulatory markers (Figure S15). Compared with the APAP+saline group, which showed elevated Bax and reduced Bcl-2 expression indicative of enhanced apoptosis, MgMn@CDs treatment dose-dependently decreased Bax while increasing Bcl-2 levels. In parallel, ferroptosis-associated GPX4 expression increased progressively with higher MgMn@CDs doses, whereas no significant change was observed in the expression of SLC7A11. These findings suggest that MgMn@CDs protect hepatocytes by suppressing mitochondrial apoptosis and restoring redox balance through ferroptosis modulation.

To evaluate the antioxidant effects of MgMn@CDs in vivo, hepatic oxidative stress biomarkers were measured following APAP administration (Fig. 6f-h). Consistent with the cellular findings, MgMn@CDs treatment significantly restored antioxidant capacity in a dose-dependent manner. The 5 and 10 mg/kg groups exhibited moderate enhancement of SOD activity and GSH levels relative to the APAP+saline group, while the 20 mg/kg group achieved values approaching those of NAC-treated mice. By contrast, the level of MDA was found to be decreased to near normal levels with the treatment of MgMn@CDs. These results demonstrate that MgMn@CDs effectively reinforce hepatic antioxidant defense systems and mitigate oxidative damage in APAP-induced liver injury.

Finally, to examine the anti-inflammatory potential of MgMn@CDs in vivo, mRNA levels of key proinflammatory cytokines (TNF-α, IL-1β, and IL-6) were measured in liver tissues using RT-qPCR. APAP overdose markedly upregulated all three cytokines, consistent with the activation of macrophage-mediated inflammatory cascades. MgMn@CDs treatment significantly reduced cytokine expression in a dose-dependent manner, with the 20 mg/kg group exhibiting mRNA levels comparable to those of the NAC-treated group (Fig. 6i-k). This indicates that MgMn@CDs attenuate APAP-induced hepatic inflammation by suppressing the transcription of proinflammatory mediators.

Taken together, MgMn@CDs exhibit potent in vivo hepatoprotective effects against APAP-induced liver injury by restoring liver function, preserving mitochondrial integrity, suppressing hepatocyte apoptosis and ferroptosis, and dampening inflammatory responses. These observations highlight the potential of MgMn@CDs as a multifunctional nanozyme for acute liver injury intervention. Further investigations will be focused on exploring the post-exposure therapeutic potential of MgMn@CDs, including delayed administration at different time windows to better simulate clinical rescue scenarios and evaluate its practical translational value.

## Conclusion

In summary, we prepared MgMn@CD nanozymes with both ultrahigh SOD-like activity (> 20000 U/mg) and satisfactory fluorescence performance (QY > 16%) via a bimetallic doping strategy. Both Mg and Mn exist in the CD framework in the form of single-atom coordination. Mg prefers to coordinate with the enol structure in CDs, thereby promoting the conversion of the keto structure to the enol structure and significantly enhancing the fluorescence properties of the CDs. In contrast, Mn tends to coordinate with the keto structure and exhibits valence variation, synergistically enhancing the SOD-like activity of the CDs. Moreover, MgMn@CD nanozymes demonstrated strong hepatoprotective activity against APAP-induced injury by efficiently scavenging ROS, restoring mitochondrial function, and suppressing apoptosis, ferroptosis and inflammation. Furthermore, the long fluorescence emission wavelength of 657 nm and high QY of MgMn@CD nanozymes provide significant potential for self-tracing in vitro and in vivo. These findings not only provide new insights into the rational design of nanozymes with both physicochemical properties and catalytic activity, but also represent a promising nanozyme platform for mitigating acute liver injury.

## Supplementary information

Supplementary material 1.

## Data Availability

No datasets were generated or analysed during the current study.

## References

[1] Maiwall R, Kulkarni AV, Arab JP, Piano S. Acute liver failure. Lancet. 2024;404(10454):789–802. 39098320 10.1016/S0140-6736(24)00693-7

[2] Zhang BQ, Zhao ZM, Chen X, Wang FQ, Luo ZW, Gu LH, et al. Kinsenoside derivatives mitigate acute liver injury in mice via MAPK pathway-mediated oxidative stress suppression. Bioorg Chem. 2025;164:108840. 40795666 10.1016/j.bioorg.2025.108840

[3] Tian Y, Zhang J, Jia Z, Pan X, Hu Z, Kang R, Zhou X, Luo L, Shen Z, Shen Q. Biomimetic mineralized mesenchymal stem cell-derived exosomes for dual modulation of ferroptosis and lactic acid-driven inflammation in acute liver injury therapy. J Colloid Interface Sci. 2025;687:489–506. 39970589 10.1016/j.jcis.2025.02.078

[4] Drug-induced liver injury. Nat Rev Dis Primers. 2019;5:59. 31439847 10.1038/s41572-019-0117-9

[5] Ren M, Lu C, Zhou M, Jiang X, Li X, Liu N. The intersection of virus infection and liver disease: a comprehensive review of pathogenesis, diagnosis, and treatment. WIREs Mechanisms of Disease. 2024;16(3):e1640. 38253964 10.1002/wsbm.1640

[6] Bunchorntavakul C, Reddy KR. Acute liver failure. Clin Liver Dis. 2017;21(4):769–92. 28987262 10.1016/j.cld.2017.06.002

[7] Sarin SK, Choudhury A. Acute-on-chronic liver failure: terminology, mechanisms and management. Nat Rev Gastroenterol Hepatol. 2016;13(3):131–49. 26837712 10.1038/nrgastro.2015.219

[8] Liu M, Huang Q, Zhu Y, Chen L, Li Y, Gong Z, et al. Harnessing reactive oxygen/nitrogen species and inflammation: nanodrugs for liver injury. Mater Today Bio. 2022;13:100215. 35198963 10.1016/j.mtbio.2022.100215 PMC8850330

[9] Yan M, Huo Y, Yin S, Hu H. Mechanisms of acetaminophen-induced liver injury and its implications for therapeutic interventions. Redox Biol. 2018;17:274–83. 29753208 10.1016/j.redox.2018.04.019 PMC6006912

[10] Yu C, Chen P, Miao L, Di G. The role of the NLRP3 inflammasome and programmed cell death in acute liver injury. Int J Mol Sci. 2023;24(4):3067. 36834481 10.3390/ijms24043067 PMC9959699

[11] Li W, Chang N, Li L. Heterogeneity and function of Kupffer cells in liver injury. Front Immunol. 2022;13:940867. 35833135 10.3389/fimmu.2022.940867 PMC9271789

[12] Hassan GS, Molina MF, Shoukry NH. The multifaceted role of macrophages during acute liver injury. Front Immunol. 2023;14:1237042. 37736102 10.3389/fimmu.2023.1237042 PMC10510203

[13] Liu X, Yu T, Hu Y, Zhang L, Zheng J, Wei X. The molecular mechanism of acute liver injury and inflammatory response induced by Concanavalin A. Mol Biomed. 2021;2(1):24. 35006454 10.1186/s43556-021-00049-w PMC8607380

[14] Sun T, Xiao S, Wang M, Xie Q, Zhang L, Gong M, et al. Reactive oxygen species scavenging nanozymes: emerging therapeutics for acute liver injury alleviation. Int J Nanomed. 2023;18:7901–22. 10.2147/IJN.S435544 PMC10750792 38148856

[15] Mooli RGR, Mukhi D, Ramakrishnan SK. Oxidative stress and redox signaling in the pathophysiology of liver diseases. Compr Physiol. 2022;12(2):3167–92. 35578969 10.1002/cphy.c200021 PMC10074426

[16] Zheng Y, Sun J, Luo Z, Li Y, Huang Y. Emerging mechanisms of lipid peroxidation in regulated cell death and its physiological implications. Cell Death Dis. 2024;15(11):859. 39587094 10.1038/s41419-024-07244-x PMC11589755

[17] Tang S-p, Mao X-l, Chen Y-h, Yan L-l, Ye L-p, Li S-w. Reactive oxygen species induce fatty liver and ischemia-reperfusion injury by promoting inflammation and cell death. Front Immunol. 2022;13:870239. 35572532 10.3389/fimmu.2022.870239 PMC9098816

[18] Garcia-Ruiz C, Fernandez-Checa JC. Mitochondrial oxidative stress and antioxidants balance in fatty liver disease. Hepatol Commun. 2018;2(12):1425–39. 30556032 10.1002/hep4.1271 PMC6287487

[19] Sadasivam N, Kim Y-J, Radhakrishnan K, Kim D-K. Oxidative stress, genomic integrity, and liver diseases. Molecules. 2022;27(10):3159. 35630636 10.3390/molecules27103159 PMC9147071

[20] Che Z, Zhou Z, Li S-Q, Gao L, Xiao J, Wong N-K. ROS/RNS as molecular signatures of chronic liver diseases. Trends Mol Med. 2023;29(11):951–67. 37704494 10.1016/j.molmed.2023.08.001

[21] Garcia-Cortes M, Ortega-Alonso A, Andradeon RJ. Spanish Grp study drug-induced L. safety of treating acute liver injury and failure. Expert Opin Drug Saf. 2022;21(2):191–203. 34254839 10.1080/14740338.2021.1955854

[22] Nilsen O, Fisher C, Warrillow S. Update on the management of acute liver failure. Curr Opin Crit Care. 2025;31(2):219–27. 39991852 10.1097/MCC.0000000000001253

[23] Fernandez J, Bassegoda O, Toapanta D, Bernal W. Acute liver failure: a practical update. JHEP Rep. 2024;6(9):101131. 39170946 10.1016/j.jhepr.2024.101131 PMC11337735

[24] Niu H, Sanabria-Cabrera J, Alvarez-Alvarez I, Robles-Diaz M, Stankeviciute S, Aithal GP, et al. Prevention and management of idiosyncratic drug-induced liver injury: systematic review and meta-analysis of randomised clinical trials. Pharmacol Res. 2021;164:105404. 33359912 10.1016/j.phrs.2020.105404

[25] Kan M, Wang Y, Cheng N, Huang K, He X. Nanozymes: new strategy for the management drug-induced acute liver injury. J Mater Chem B. 2025;13(30):9023–42. 40637149 10.1039/d5tb00448a

[26] Licata A, Minissale MG, Stankeviciute S, Sanabria-Cabrera J, Lucena MI, Andrade RJ, et al. N-acetylcysteine for preventing acetaminophen-induced liver injury: a comprehensive review. Front Pharmacol. 2022;13:828565. 36034775 10.3389/fphar.2022.828565 PMC9399785

[27] Tang G, He J, Liu J, Yan X, Fan K. Nanozyme for tumor therapy: surface modification matters. Exploration. 2021;1(1):75–89. 37366468 10.1002/EXP.20210005 PMC10291575

[28] Zhao Y, Zhang Z, Pan Z, Liu Y. Advanced bioactive nanomaterials for biomedical applications. Exploration. 2021;1(3):20210089. 37323697 10.1002/EXP.20210089 PMC10191050

[29] Yang J, Zhang R, Zhao H, Qi H, Li J, Li J-F, et al. Bioinspired copper single-atom nanozyme as a superoxide dismutase-like antioxidant for sepsis treatment. Exploration. 2022;2(4):20210267. 37325607 10.1002/EXP.20210267 PMC10191017

[30] Lee J, Liao H, Wang Q, Han J, Han J-H, Shin HE, et al. Exploration of nanozymes in viral diagnosis and therapy. Exploration. 2022;2(1):20210086. 37324577 10.1002/EXP.20210086 PMC10191057

[31] Huang Y, Ren J, Qu X. Nanozymes: classification, catalytic mechanisms, activity regulation, and applications. Chem Rev. 2019;119(6):4357–412. 30801188 10.1021/acs.chemrev.8b00672

[32] Li X, Wang L, Du D, Ni L, Pan J, Niu X. Emerging applications of nanozymes in environmental analysis: opportunities and trends. TrAC Trends Anal Chem. 2019;120:115653.

[33] Oro D, Yudina T, Fernandez-Varo G, Casals E, Reichenbach V, Casals G, et al. Cerium oxide nanoparticles reduce steatosis, portal hypertension and display anti-inflammatory properties in rats with liver fibrosis. J Hepatol. 2016;64(3):691–8. 26519601 10.1016/j.jhep.2015.10.020

[34] Ibrahim HG, Attia N, Hashem FEZA, El Heneidy MAR. Cerium oxide nanoparticles: in pursuit of liver protection against doxorubicin-induced injury in rats. Biomed Pharmacother. 2018;103:773–81. 29684856 10.1016/j.biopha.2018.04.075

[35] Soh M, Kang DW, Jeong HG, Kim D, Kim DY, Yang W, et al. Ceria–zirconia nanoparticles as an enhanced multi-antioxidant for sepsis treatment. Angew Chem Int Ed. 2017;56(38):11399–1403. 10.1002/anie.201704904 28643857

[36] Feng Q, Xu H, Pan X, Geng S, Qian H, Wang C, et al. Antioxidation and anti-inflammatory activity of prussian blue nanozymes to alleviate acetaminophen-induced acute liver injury. ACS Appl Nano Mater. 2023;6(10):8468–81.

[37] Bai H, Kong F, Feng K, Zhang X, Dong H, Liu D, et al. Prussian blue nanozymes prevent anthracycline-induced liver injury by attenuating oxidative stress and regulating inflammation. ACS Appl Mater Interfaces. 2021;13(36):42382–95. 34473471 10.1021/acsami.1c09838

[38] Chen C, Wu H, Li Q, Liu M, Yin F, Wu M, et al. Manganese prussian blue nanozymes with antioxidant capacity prevent acetaminophen-induced acute liver injury. Biomater Sci. 2023;11(7):2348–58. 36722889 10.1039/d2bm01968j

[39] Lu Y, Pan X, Cao C, Fan S, Tan H, Cui S, et al. MnO2 coated mesoporous PdPt nanoprobes for scavenging reactive oxygen species and solving acetaminophen-induced liver injury. Adv Healthc Mater. 2023;12(22):2300163. 10.1002/adhm.202300163 37184887

[40] Liu C, Fan W, Cheng W-X, Gu Y, Chen Y, Zhou W, Yu X-F, Chen M, Zhu M, Fan K, et al. Red Emissive Carbon Dot Superoxide Dismutase Nanozyme for Bioimaging and Ameliorating Acute Lung Injury. Adv Funct Mater. 2023;33(19):2213856.

[41] Gao W, He J, Chen L, Meng X, Ma Y, Cheng L, et al. Deciphering the catalytic mechanism of superoxide dismutase activity of carbon dot nanozyme. Nat Commun. 2023;14(1):160. 36631476 10.1038/s41467-023-35828-2 PMC9834297

[42] Ma Y, Zhao J, Cheng L, Li C, Yan X, Deng Z, Zhang Y, Liang J, Liu C, Zhang M. Versatile carbon dots with superoxide dismutase-like nanozyme activity and red fluorescence for inflammatory bowel disease therapeutics. Carbon. 2023;204:526–37.

[43] Yan Z, Zhang Y, Chen Q, Li J, Ning X, Bai F, Wang Y, Liu X, Liu Y, Zhang M, et al. Carbon dot superoxide dismutase nanozyme enhances reactive oxygen species scavenging in diabetic skin wound repair. J Adv Res. 2025;79:691–706. 40154736 10.1016/j.jare.2025.03.049 PMC12766211

[44] Zhang Y, Shi J, Fang X, Li J, Fan X, Wang K, et al. Mg/Mn co-doped carbon dot nanozyme with high superoxide dismutase-like activity and fluorescence for transdermal therapy of atopic dermatitis. Chem Eng J. 2025;523:168610.

[45] Li J, Shi J, Fang X, Zhang Y, Xie Q, Danzeng Q, et al. Tuning the excited-state intramolecular proton transfer in carbon dots via coordination with metal ion. Inorg Chem. 2025;64(15):7706–15. 40202949 10.1021/acs.inorgchem.5c00731

[46] Shi J, Li J, Li X, Zhang Y, Hu J, Ning Y, et al. Coordination of Mg(II) enhancing photoinduced oxidase-like activity of carbon dots for efficient degradation of organic dyes. Chem Mater. 2025;37(6):2290–301.

[47] Shi J, Zhang Y, Fang X, Fan X, Li J, Zhou C-H, et al. Photoswitchable antioxidant and prooxidant activities of Mg-doped carbon dot nanozymes as antibacterial and anti-inflammatory agents. ACS Applied Materials & Interfaces. 2025;17(18):26467–79. 40293447 10.1021/acsami.5c05025

[48] Pan L, Sun S, Zhang L, Jiang K, Lin H. Near-infrared emissive carbon dots for two-photon fluorescence bioimaging. Nanoscale. 2016;8(39):17350–6. 27714173 10.1039/c6nr05878g

[49] Li J, Xiang Y, Zhang Q, Wu W, Gao G, Zhou CH, Wu K, Xia Z, Pang DW, Liu C. Rational design of carbon dot‐supported Mn─Zn bimetallic single‐atom nanozymes for enhancing SOD‐like activity and fluorescence. Adv Funct Mater. 2026:e31973. 10.1002/adfm.202531973

[50] Trujillo-Hernandez JA, Levine RL. Response to oxidative stress of AML12 hepatocyte cells with knockout of methionine sulfoxide reductases. Free Radic Biol Med. 2023;205:100–6. 37290581 10.1016/j.freeradbiomed.2023.05.028 PMC11626390

[51] Liao J, Lu Q, Li Z, Li J, Zhao Q, Li J. Acetaminophen-induced liver injury: molecular mechanism and treatments from natural products. Front Pharmacol. 2023;14:1122632. 37050900 10.3389/fphar.2023.1122632 PMC10083499

[52] Malla S, Neupane R, Sood S, Hussein N, Abou-Dahech M, Terrero D, et al. Mitochondria as regulators of nonapoptotic cell death in cancer. MedComm. 2025;6(8):e70244. 40703196 10.1002/mco2.70244 PMC12284444

[53] Mursal M, Hasan I, Tiwari B, Srivastava RK, Yadav G, Fatima G. Disruptions in nitric oxide homeostasis, lipid peroxidation-derived oxidative stress, and antioxidant defense mechanisms in spinal cord injury: elucidating biomolecular correlates of disease severity. Mol Biol Rep. 2025;52(1):969. 41026327 10.1007/s11033-025-11091-0

[54] Hionides-Gutierrez A, Goikoetxea-Usandizaga N, Sanz-Garcia C, Martinez-Chantar ML, Cubero FJ. Novel emerging mechanisms in acetaminophen (APAP) hepatotoxicity. Liver Int. 2025;45(4):e16167. 39548712 10.1111/liv.16167

[55] Ruan H, Wang L, Wang J, Sun H, He X, Li W, et al. Sika deer antler protein against acetaminophen-induced oxidative stress and apoptosis in HK-2 cells via activating Nrf2/keap1/HO-1 pathway. J Food Biochem. 2019;43(12):e13067. 31599006 10.1111/jfbc.13067

[56] Jaeschke H, Ramachandran A. The role of oxidant stress in acetaminophen-induced liver injury. Curr Opin Toxicol. 2020;20–21:9–14. 32309680 10.1016/j.cotox.2020.03.003 PMC7164773

