# Supplementary material for: Mg/Mn bi-single-atom carbon dot nanozymes with enhanced SOD-like activity and fluorescence for hepatoprotection
Source: J Nanobiotechnology. 2026 Apr 21;24:696. doi: 10.1186/s12951-026-04464-5 (PMC13393799; doi:10.1186/s12951-026-04464-5)
Supplement: Supplementary file 1 — Supplementary material 1. [file 12951_2026_4464_MOESM1_ESM.docx]

**Supplementary Information**

**Mg/Mn bi-single-atom carbon dot nanozymes with enhanced SOD-like activity and fluorescence for hepatoprotection**

Xuejiao Wang,^a^ Yu Zhang,^b^ Jing Li,^b^ Hongjing Cheng,^c^ Hanyue Qiu,*^,b^ Jian Jiao,*^,a^ Cui Liu*^,b^

^a^ Department of Digestive, China-Japan Union Hospital of Jilin University, Changchun 130033, P. R. China. E-mail: jjian@jlu.edu.cn

^b^ Chongqing Key Laboratory of Natural Product Synthesis and Drug Research, Innovative Drug Research Center, School of Pharmaceutical Sciences, Chongqing University, Chongqing, 400044, P. R. China. E-mail: hanyue.qiu@cqu.edu.cn; liucui@cqu.edu.cn

^c^ Department of Pathology, China-Japan Union Hospital of Jilin University, Changchun 130033, P. R. China

**Methods**

**Chemicals and materials**

Glutathione, formamide, and manganese(III) acetylacetonate were purchased from Shanghai Aladdin Biochemical Technology Co., Ltd. Magnesium chloride hexahydrate was purchased from Chengdu Kelong Chemical Co., Ltd. Terephthalic acid (TA) was purchased from Shanghai Titan Technology Co., Ltd. Acetaminophen was purchased from MedChemExpress LLC. SOD Assay Kit-WST (S311) was purchased from Dojindo Beijing Co., Ltd. ABTS Assay Kit (BC4775) was purchased from Beijing Solarbio Science & Technology Co., Ltd. MTT was purchased from Labgic Technology Co., Ltd. (Beijing, China). Annexin V-FITC Cell Apoptosis Detection Kit (C1062L), Mitochondrial Membrane Potential Detection Kit (JC-1) (C2006), Reactive Oxygen Species Detection Kit (S0033S), Total Glutathione Assay Kit (S0052), and Lipid Peroxidation (MDA) Assay Kit (S0131S) were purchased from Beyotime Biotechnology Co., Ltd (Shanghai, China). The primary antibodies against Bcl-2 (WL01556), Bax (WL01637), GAPDH (WL01114), and the secondary antibody (WLA023a) were purchased from Wanlei Bio (Shenyang, China). The primary antibodies against GPX-4 (F1580) and SLC7A11 (F0517) were purchased from Selleck Chemicals (China). ExonScript RT Mix (with dsDNase) and SYBRPrime qPCR Set were purchased from Baoguang Biotechnology Co., Ltd.

**Instrumentation**

The morphology of MgMn@CDs was acquired by the FEI Tecnai G2 F20 transmission electron microscope (acceleration voltage 300 kV ). The high-angle annular dark-field scanning transmission electron microscope (HAADF-STEM) images and the corresponding atomic-resolution energy-dispersive X-ray spectroscopy element mappings were recorded by the JEM-ARM300F in situ double aberration-corrected transmission electron microscope (JEOL, Japan) operated at 300 kV. The fluorescence emission spectra were recorded by the F-4600 Fluorescence spectrometer (HITACHI, Japan). The UV-vis absorption spectra were obtained by the UH-5300 Ultraviolet-visible spectrometer (HITACHI, Japan). The proton magnetic resonance (^1^H-NMR) spectra were obtained by the Agilent DD2 600 MHz nuclear magnetic resonance spectrometer (Agilent, USA, D_2_O as the solvent). The Fourier transform infrared (FT-IR) spectra were recorded by the Bruker Tensor 27 FT-IR spectrometer (Bruker Corporation, USA). The fluorescence lifetimes and absolute quantum yields were recorded by the FLS1000 spectrometer (Edinburgh Instruments, UK). The X-ray photoelectron spectroscopy (XPS) spectra were obtained by the Thermo ESCALAB 250Xi X-ray photoelectron spectrometer (ThermoFisher Scientific, USA). The metal loading of metal-doped CDs was obtained by the iCAP6300 inductively coupled plasma optical emission spectrometer (ThermoFisher Scientific, USA). X-ray absorption spectroscopy (XAS) measurements of the Mg K-edge were obtained at I06 of Diamond Light Source (UK). X-ray absorption spectroscopy (XAS) measurements of the Mn K-edge were obtained at B18 of Diamond Light Source (UK). The absorbance values were obtained using the SpectraMax i3x microplate reader (Molecular Devices, USA). Flow cytometry data were acquired through the CytoFLEX analytical flow cytometer (Beckman Coulter, USA). Cellular fluorescence images were acquired by the Ti-S inverted fluorescence microscope (Nikon, Japan). Histological slides were imaged by the Leica DM6 upright fluorescence microscope (Leica, Germany). Protein imaging was performed using the Bio-Rad ChemiDoc Touch chemiluminescence imaging system (Bio-Rad Laboratories, USA). Real-time fluorescent quantitative PCR was performed using the CFX96 Touch (Bio-Rad Laboratories, USA). Fluorescence imaging in mice was obtained using the In Vivo Small Animal Optical Imaging System (IVIS Lumina III, PerkinElmer, USA).

**Synthesis of MgMn@CDs**

MgMn@CDs were synthesized using a one-step solvothermal method, according to our previous work.^1^ Briefly, 20 mg of magnesium chloride hexahydrate, 10 mg of manganese (III) acetylacetonate, along with 0.7 g of reduced glutathione were added to 70 mL of formamide. The mixture was transferred into a Teflon autoclave and reacted at 160 ℃ for 8 h. The resulting solution was cooled to room temperature and dialyzed for 7 days using a 3500-Da dialysis bag. The solution was concentrated and then freeze-dried to obtain MgMn@CDs. CDs without metal doping were prepared by the same method without adding metal salts. Similarly, Mg-CDs were prepared by adding 20 mg of magnesium chloride hexahydrate, while Mn-CDs were prepared by adding 10 mg of manganese (III) acetylacetonate.

**SOD-like activity and O_2_^•−^ scavenging activity of MgMn@CDs**

The SOD-like activity of MgMn@CDs was detected using a commercial SOD Assay Kit (S311, Dojindo Molecular Technolo gies, Inc.). This method leverages WST-1 ((2-(4-Iodophenyl)-3-(4-nitrophenyl)-5-(2,4-disulfophenyl)-2H-tetrazolium, monosodium salt)), a highly water-soluble tetrazolium salt, which interacts with superoxide anions to generate a formazan dye exhibiting absorbance peak at 450 nm. The extent of WST-1 reduction by superoxide anions directly correlates with xanthine oxidase activity and is suppressed by SOD. The inhibition rate of SOD (%) was calculated according to the following equation. Additionally, the O_2_^•−^ scavenging capacity of MgMn@CDs can be evaluated by monitoring the change in UV-vis absorption intensity of the reaction system at approximately 450 nm before and after the addition of MgMn@CDs.

The inhibition rate of SOD (%) = $\frac{\text{（}\text{A}_{\text{Control}\text{ }\text{1}}-\text{A}_{\text{Control}\text{ }\text{3}}\text{)}-(\text{A}_{\text{Sample}}-\text{A}_{\text{Control}\text{ }\text{2}}\text{)}}{\text{(}\text{A}_{\text{Control}\text{ }\text{1}}-\text{A}_{\text{Control}\text{ }\text{3}}\text{)}}\text{×100}$

Control 1: coloring without inhibitor, Control 2: sample blank, Control 3: reagent blank.

According to the instruction of the commercial SOD assay kit (WST-1 method, Dojondo, No. S311), 1 Unit (U) of SOD is defined as the amount of enzyme that inhibits 50% of the reduction reaction between WST-1 and superoxide anions in 20 μL of sample solution. Thus, the specific activity of CDs was calculated using the IC_50_ (50% inhibitory concentration). To determine the IC_50_, a curve of inhibition rate against the final concentration of CDs was plotted. Nonlinear regression analysis was then performed by selecting the model of [Inhibitor] vs. normalized response-Variable slope. The calculation formula of SOD specific activity is as follows:

$$SOD specific activity=\frac{1000}{\mathrm{IC}_{50}\times V}$$

IC_50_: half maximal inhibitory concentration; V: total volume of the reaction system.

**•OH scavenging activity of MgMn@CDs**

The generation of ⋅OH is achieved by irradiating H_2_O_2_ under ultraviolet light. Terephthalic acid (TA) is used as a probe and reacts with ⋅OH to form 2-hydroxyterephthalic acid, which exhibits a characteristic fluorescence peak at approximately 430 nm. The ⋅OH scavenging capacity of MgMn@CDs can be evaluated by comparing the variation in fluorescence intensity around 430 nm before and after the addition of MgMn@CDs. 2 mM H_2_O_2_, 5 mM TA, and MgMn@CDs at different concentrations (0, 10, 20, and 40 μg/mL) were mixed and irradiated under UV light for 15 min. Subsequently, the fluorescence emission spectra of all groups were recorded.

**ABTS****^•+^ scavenging activity of MgMn@CDs**

The ABTS^•+^ scavenging capacity of MgMn@CDs was determined using a commercial assay kit (BC4775, Beijing Solarbio Science & Technology Co., Ltd.). ABTS is oxidized to form stable blue-green ABTS**^•+^**, which exhibits a characteristic absorption at 405 nm. When antioxidant samples are added, they react with ABTS**^•+^**, resulting in the discoloration of the reaction system and a decrease in absorbance. The ABTS**^•+^** scavenging capacity of MgMn@CDs can be evaluated by measuring the degree of absorbance decrease.

***In vitro* cytotoxicity study**

The cytotoxicity of MgMn@CDs was evaluated using the MTT assay. AML-12 cells were seeded into 96-well culture plates at a density of 1 × 10^4^ cells per well and cultured at 37 °C for 24 h. Subsequently, cells were treated with different concentrations of MgMn@CDs for 24 h. After the cells were incubated with 5 mg/mL MTT for another 4 h, the suspension was discarded and the dark blue formazan crystals were dissolved in DMSO. Then, the absorbance at 490 nm was measured using a Spectramax microplate reader. In all experiments, three replicate wells were used for each drug concentration. Each assay was performed at least three times.

**Intracellular ROS detection**

AML-12 cells were seeded into a 6-well culture plate at a density of 2 × 10^5^ cells per well. After treatment, intracellular production of ROS was detected based on the oxidation of the cell-permeable dye 2*'*,7*'*-dichlorofluorescin diacetate (DCFH-DA). DCFH‑DA was diluted 1:1000 in serum‑free culture medium. Subsequently, the diluted DCFH-DA solution (10 μM) was added to each well and incubated at 37 °C for 30 min. After incubation, the cells were washed three times with PBS. ROS generation was observed under a fluorescence microscope.

**Mitochondrial membrane potential evaluation**

Mitochondrial transmembrane potential (∆Ψ_m_) was detected using a JC-1 mitochondrial membrane potential assay kit. After the treatment, the cells were incubated with 5 μg/mL JC-1 (5,5,6,6-tetrachloro-1,1,3,3-tetraethylbenzimidazolylcarbocyanine iodide) at 37 °C for 20 min, and then washed three times with PBS and resuspended in fresh serum-free medium. Samples were visually assessed using a fluorescence microscope.

**Apoptosis assay**

AML-12 cells in the logarithmic growth phase were seeded in a 6-well plate at a density of 2 × 10^5^ per well. After 24 h of treatment, the cells were trypsinized, washed three times with PBS and centrifuged at 2000 rpm for 5 min. The cell pellets were gently resuspended in 500 μL of staining solution (containing 5 μL AnnexinV-FITC and 5 μL PI in binding buffer), mixed gently and incubated for 15 min at room temperature in the dark. The cells were then analyzed using a FACSCalibur flow cytometer.

***In vivo* biocompatibility and pharmacokinetic evaluation**

To assess the *in vivo* biocompatibility and pharmacokinetic profiles of MgMn@CDs, ICR mice aged 6-8 weeks were intraperitoneally injected with MgMn@CDs at a single dose of 20 mg/kg (n=6). A control group received an intraperitoneal injection of saline. For pharmacokinetic analysis, blood samples were collected from the mice at 10, 15, 20, 30, 40, 50, 60, 120, 240, and 480 minutes post-injection. Additionally, blood samples were collected at 30 days post-injection for serum biochemistry testing, including the measurement of a panel of hepatic and renal function markers. Subsequently, the mice were sacrificed, and major organs, including the heart, liver, spleen, lung, and kidney, were harvested for H&E staining.

***In vivo* and *ex vivo* fluorescence imaging of MgMn@CDs in mice**

The abdominal hair of ICR mice was removed. Then, MgMn@CDs (10 mg/kg) were administered intraperitoneally into the mice. The control group received MgMn@CDs in saline, while treatment group received MgMn@CDs with APAP. *In vivo* imaging was performed at each time point. For *ex vivo* imaging, at the specified time point after treatment, the mice were sacrificed humanely. Subsequently, the major organs, including the heart, liver, spleen, lung, and kidney, were carefully dissected and immediately subjected to *ex vivo* imaging to accurately evaluate the distribution and accumulation of MgMn@CDs.

**Animal model and experimental design**

After a 7-day acclimation period, the mice were randomly divided into six groups: a normal control group, an APAP-induced acute liver injury model group, a positive control group (NAC, 150 mg/kg), and three treatment groups that receiving MgMn@CDs at 5, 10, or 20 mg/kg. All mice were fasted overnight. Two hours prior to APAP challenge, the positive control group was administered NAC (150 mg/kg, i.p.), and the treatment groups received MgMn@CDs (5, 10, or 20 mg/kg, i.p.), while the normal control and model groups were given saline vehicle. Subsequently, mice in the model, positive control, and treatment groups received an intraperitoneal injection of APAP (300 mg/kg) to induce liver injury; the normal control group received an equivalent volume of saline. Blood samples were collected from the mice 24 hours after APAP exposure for serum biochemical analysis, including hepatic function markers (AST and ALT). Finally, the mice were euthanized humanely, and liver tissues were harvested for further analysis.

**Transmission electron microscopy analysis**

Liver tissues were collected from euthanized mice and cut into small cubes. Tissues were fixed in 2.5% glutaraldehyde in 0.1 M phosphate buffer at 4 ℃, then post-fixed with 1% OsO_4_ for 1 h at room temperature. After dehydration with graded ethanol solutions, tissues were embedded in epoxy resin. Ultrathin sections (70 nm) were cut, stained with lead citrate and uranyl acetate, and imaged using a Hitachi H-7650 TEM at 100 kV. The experiment was repeated three times with consistent results.

**Western blotting** **analysis**

Cells in 6-well plates were rinsed twice with cold PBS and lysed in RIPA lysis buffer containing a protease inhibitor (PMSF) at a 1:100 dilution on ice for 30 min. Insoluble debris was removed by centrifugation at 12000 × g for 10 min at 4 ℃, and protein concentrations were measured using a Pierce BCA protein assay kit. Proteins were separated by sodium dodecyl sulfate-polyacrylamide gel electrophoresis (SDS-PAGE) and transferred onto polyvinylidene difluoride (PVDF) membranes. Membranes were blocked with 5% skim milk in TBST, incubated with primary antibodies overnight at 4 ℃, washed five times with TBST (5 min each), and then incubated with HRP-conjugated goat anti-rabbit IgG for 1 h at room temperature. Protein bands were visualized using an ECL kit and imaged with a ChemiDoc system.

**RNA extraction and qRT-PCR analysis**

Liver tissues or cultured cells were homogenized in TRIzol (1 mL). After standing for 5 min, chloroform was added, and the mixture was vigorously shaken and incubated for 5 min. After centrifugation at 12,000 × g at 4 °C for 15 min, the supernatant was transferred into a new tube. Then, isopropanol was added, followed by gently shaking and incubated at room temperature for 10 min. The precipitate was collected by centrifugation at 12,000 × g, 4 °C for 10 min, resuspended in pre-cooled 75% ethanol, and centrifuged again. The RNA pellets was air-dried and dissolved in 50 μL DEPC-treated water. The purity and concentration of total RNA were determined. Total RNA was reverse-transcribed to cDNA. Quantitative real-time PCR was performed in triplicate with a SYBR Green Premix qPCR kit on the CFX96 Touch. Relative mRNA expression levels were calculated using 2^-△△Ct^ method, with GAPDH as the internal reference.

**Statistical analysis**

Quantitative data are displayed as means ± SD. Statistical differences were determined using Student's t-test for two groups or one-way analysis of variance (ANOVA) for three or more groups. Statistical analyses were performed using GraphPad Prism 8. Statistical significance was denoted as *p < 0.05, **p < 0.01, ***p < 0.001, ****p < 0.0001.

**Supplementary Figures**


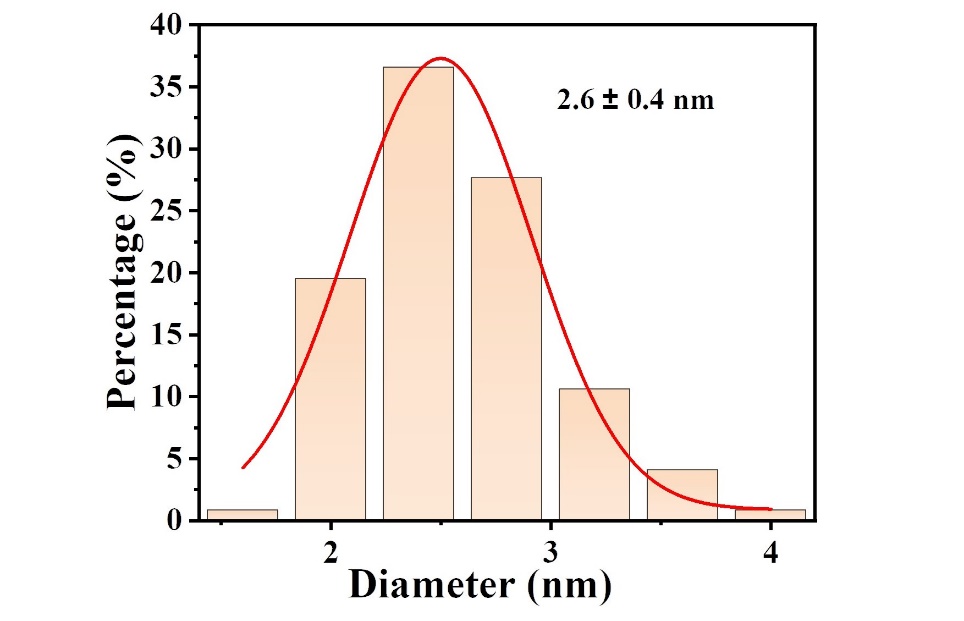


**Fig. S1.** Histogram of particle size distribution of MgMn@CDs.

*
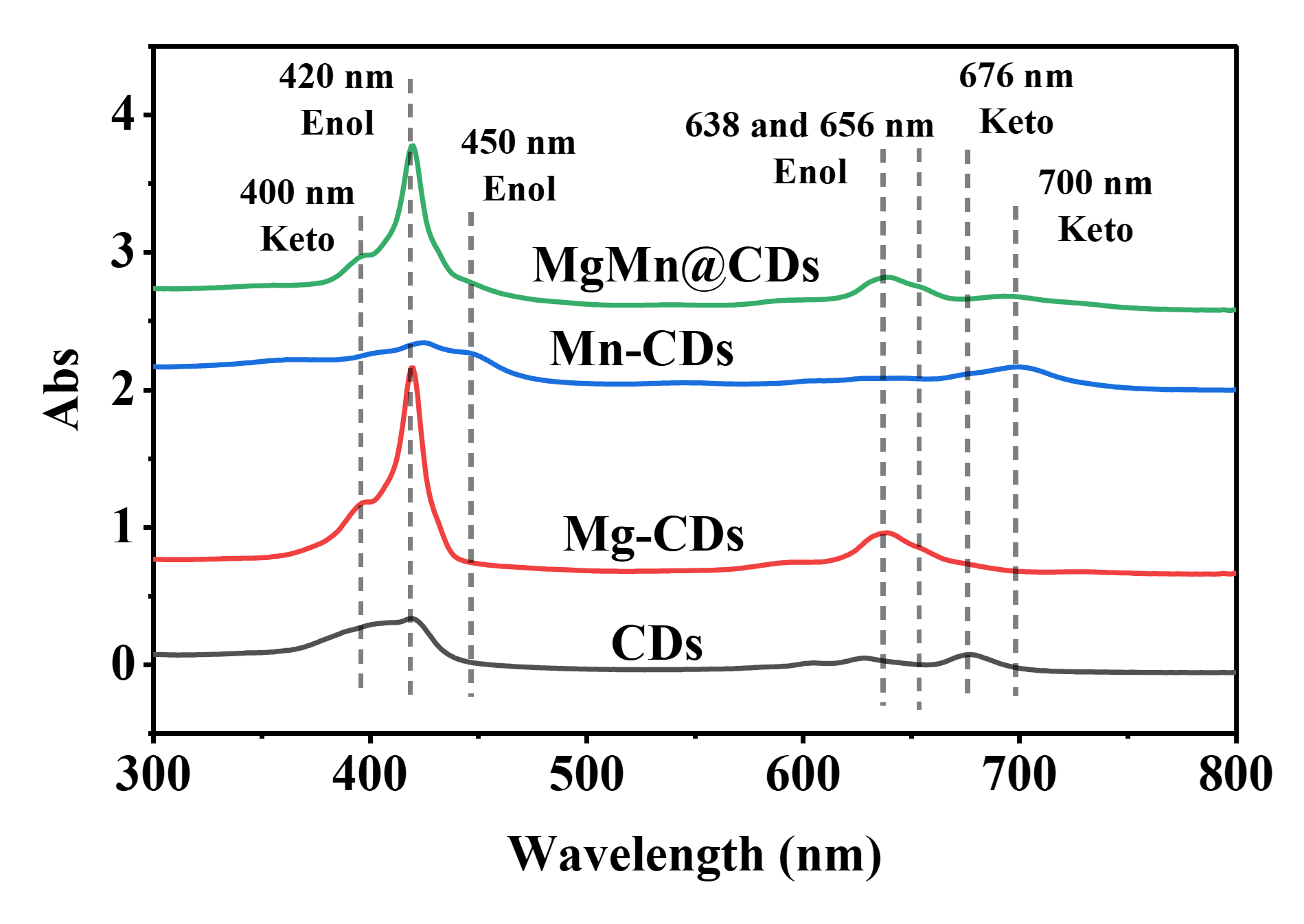
*

**Fig. S2**. The UV-vis absorption spectra of CDs, Mg-CDs, Mn-CDs, and MgMn@CDs.


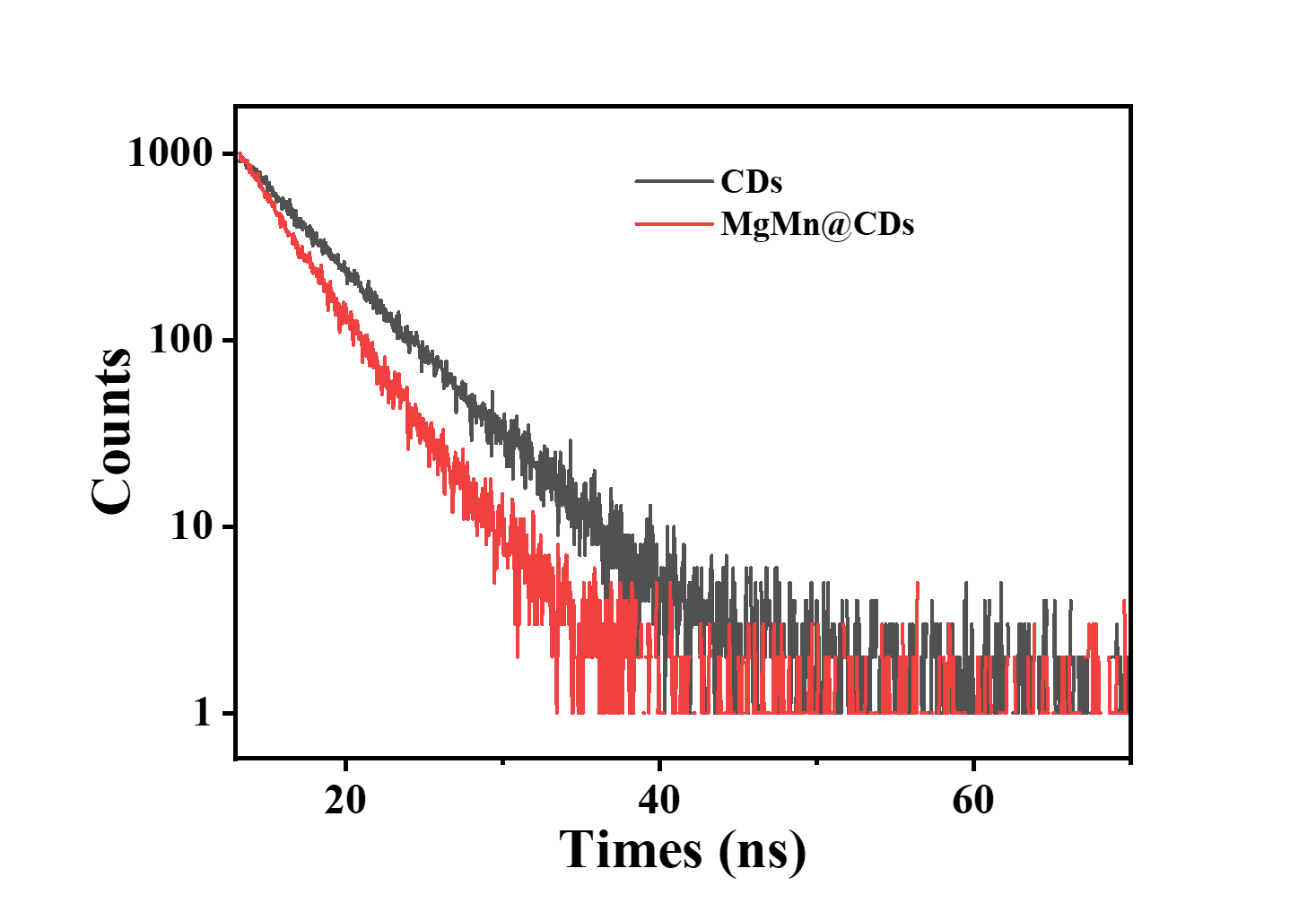


**Fig. S3.** Fluorescence decay curves of CDs and MgMn@CDs.


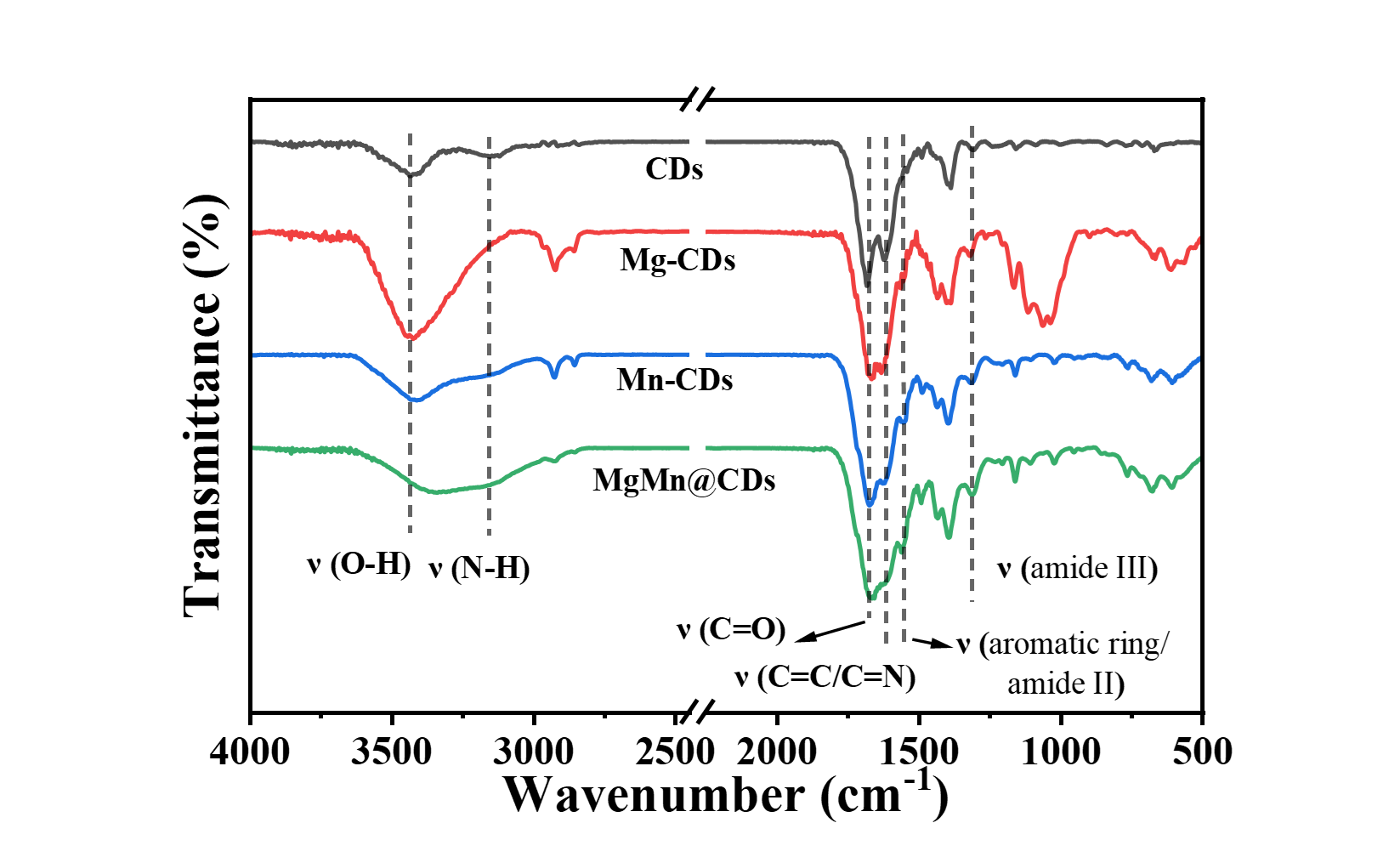


**Fig. S4.** The FT-IR spectra of CDs, Mg-CDs, Mn-CDs, and MgMn@CDs.


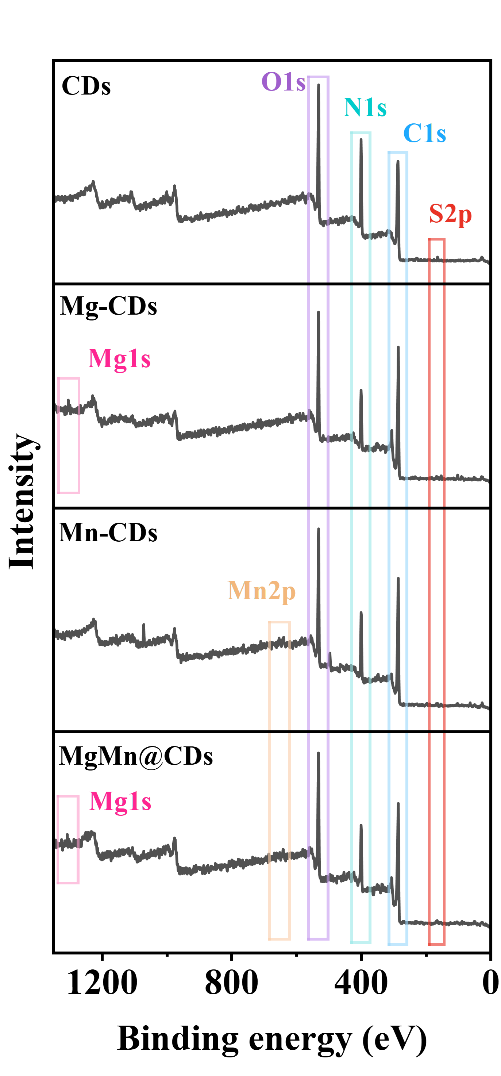


**Fig. S5.** XPS survey spectra of CDs, Mg-CDs, Mn-CDs and MgMn@CDs.


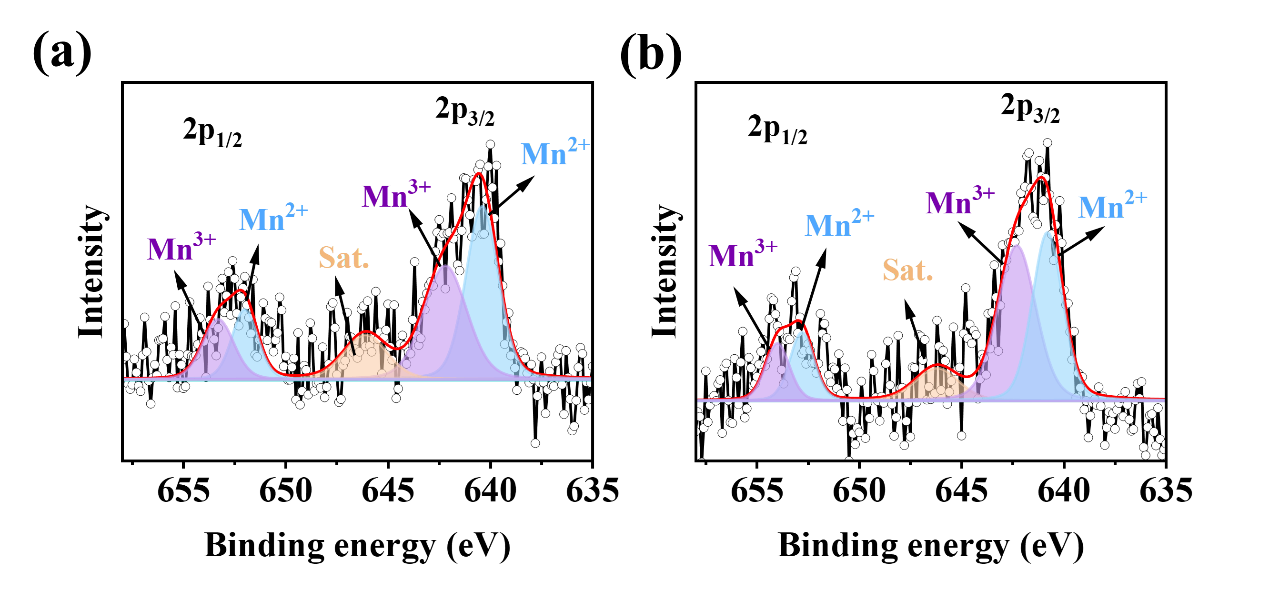


**Fig. S6.** HR Mn 2p spectra of (a) Mn-CDs and (b) MgMn@CDs.


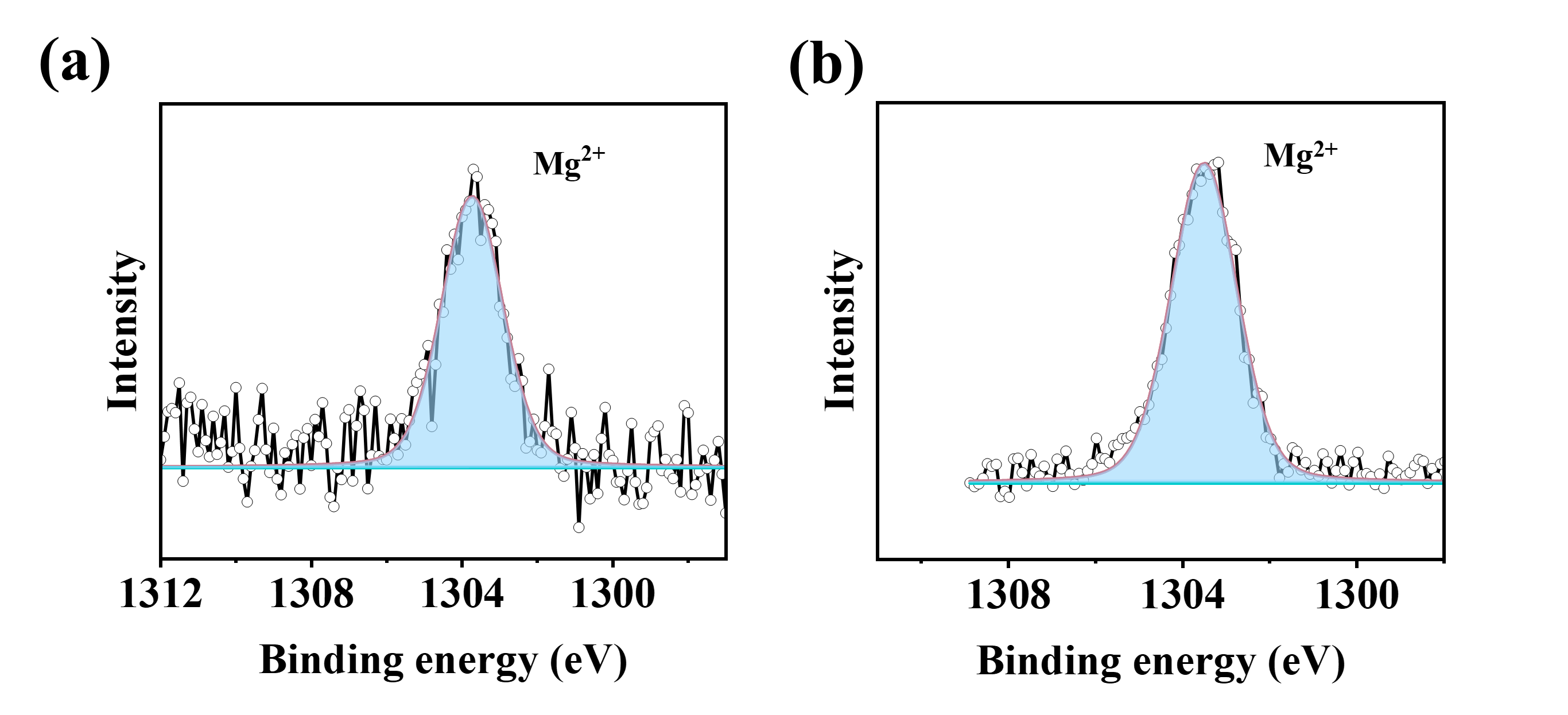


**Fig. S7.** HR Mg 1s spectra of (a) Mg-CDs and (b) MgMn@CDs.


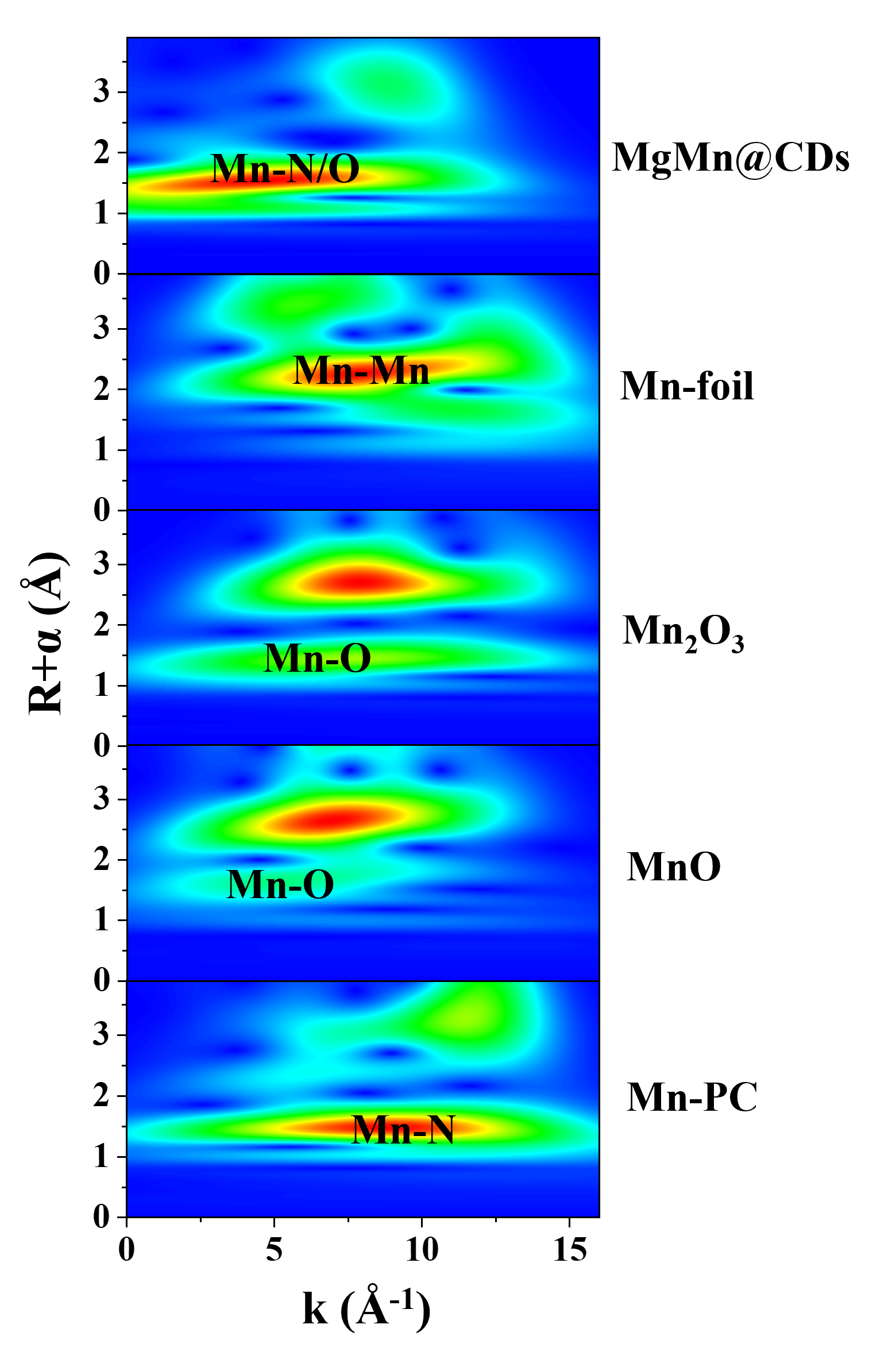


**Fig. S8.** Mn K-edge WT-EXAFS curves of MgMn@CDs and reference samples.


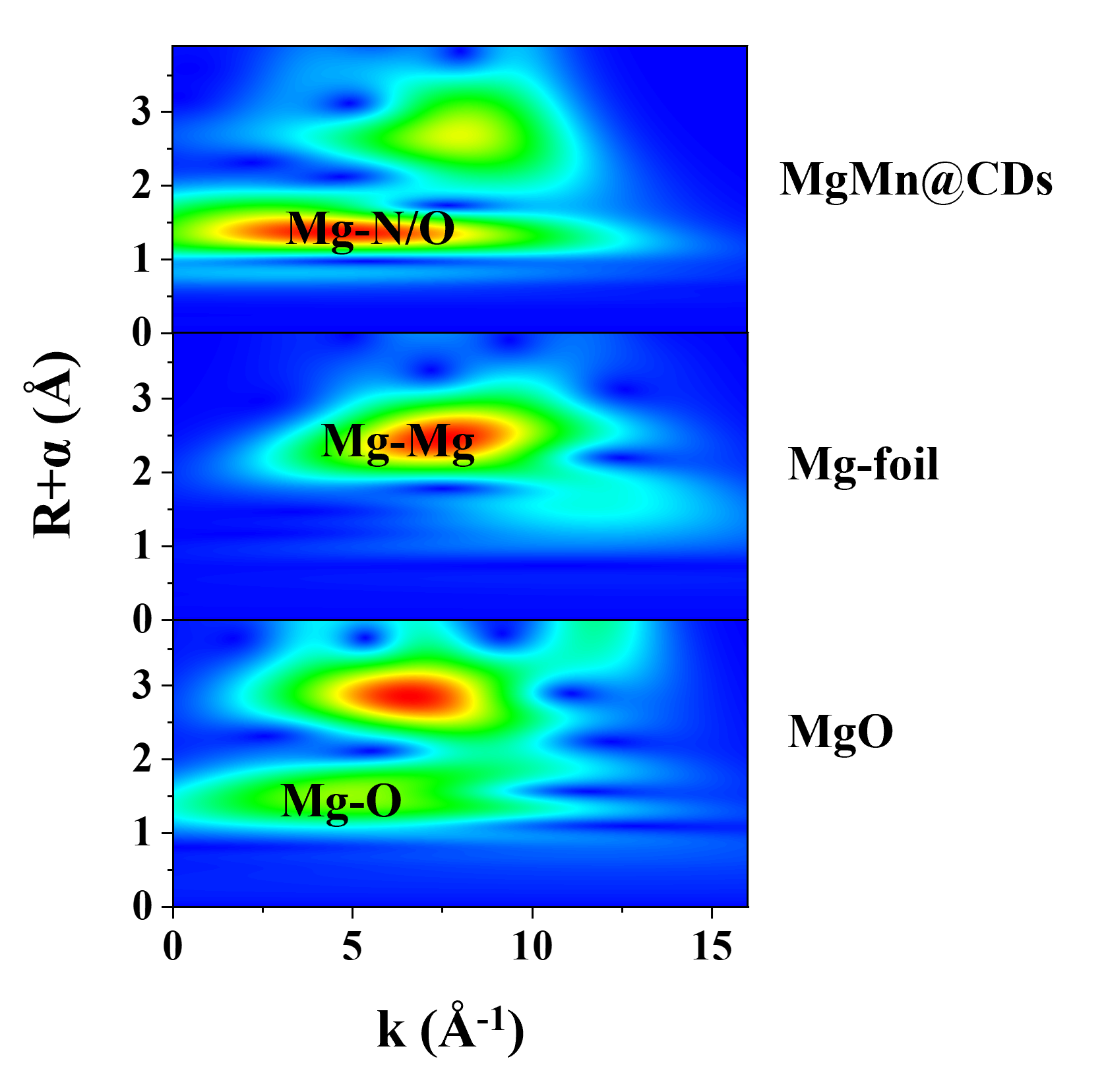


**Fig. S9.** Mg K-edge WT-EXAFS curves of MgMn@CDs and reference samples.


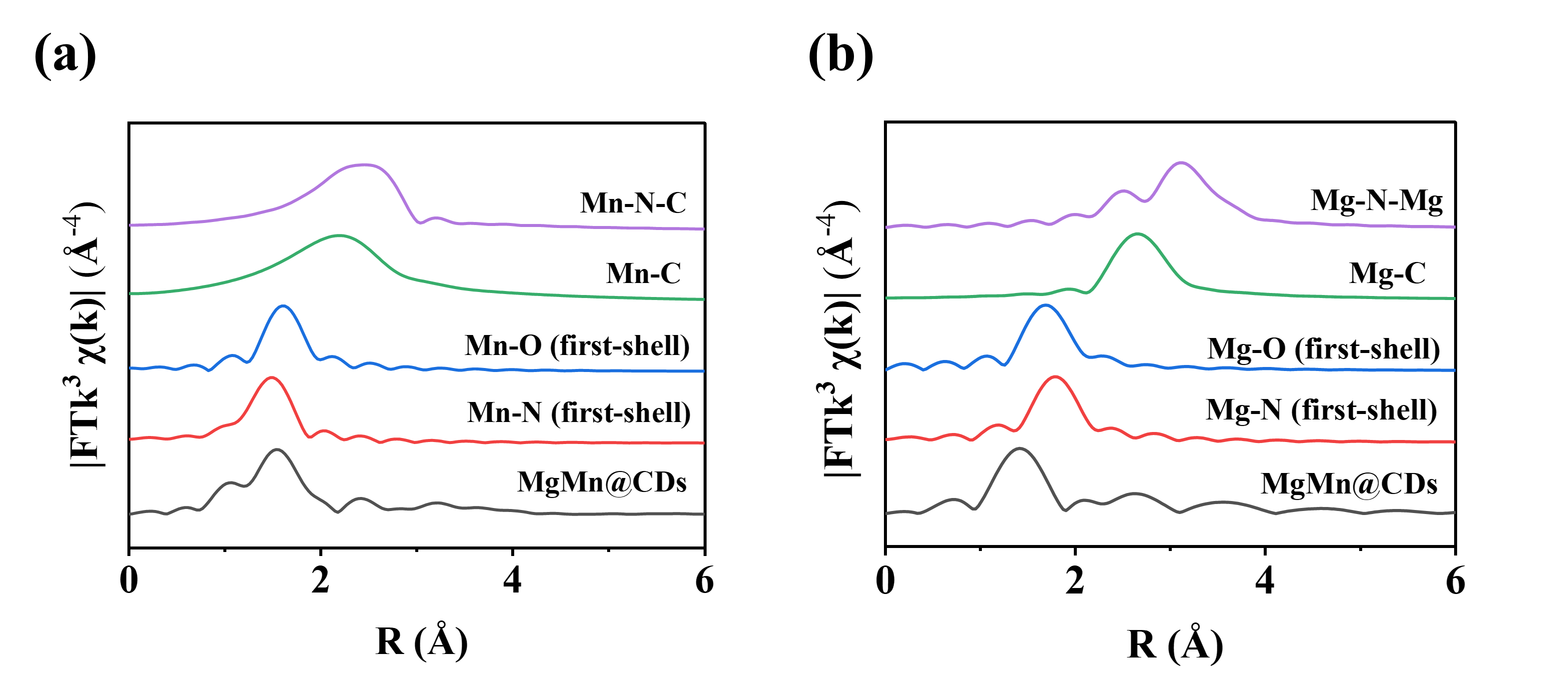


**Fig. S10.** The (**a**) Mn and (**b**) Mg K-edge FT-EXAFS of MgMn@CDs with different scattering path.


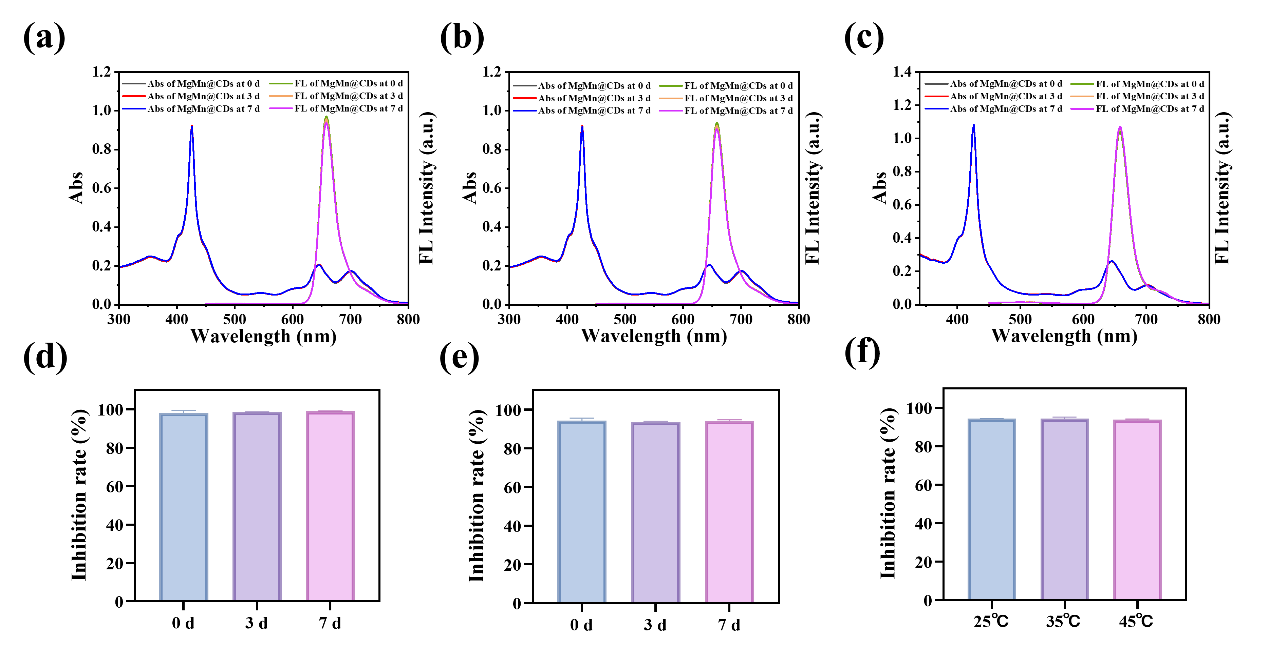


**Fig. S11.** Stability of optical properties of MgMn@CDs after incubation in (a) water, (b) PBS, and (c) FBS for 0, 3, and 7 days (final concentration of 10 μg/mL). Stability of SOD-like activity of MgMn@CDs after incubation in (d) PBS and (e) FBS for 0, 3, and 7 days (final concentration of 0.65 μg/mL). (f) Stability of SOD-like activity of MgMn@CDs at 25 °C, 35 °C, and 45 °C (final concentration of 0.65 μg/mL).


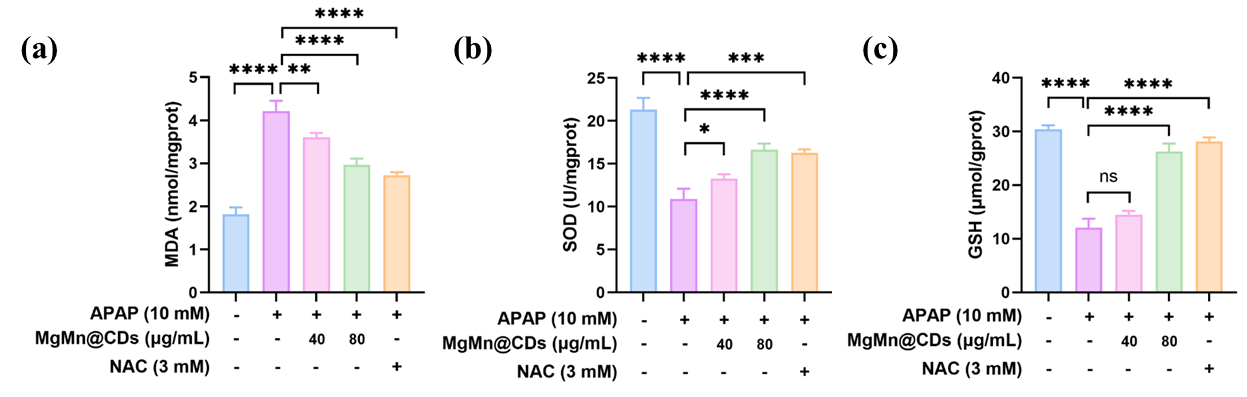


**Fig. S12.** Levels of oxidative stress biomarkers: (**a**) MDA, (**b**) SOD and (**c**) GSH.


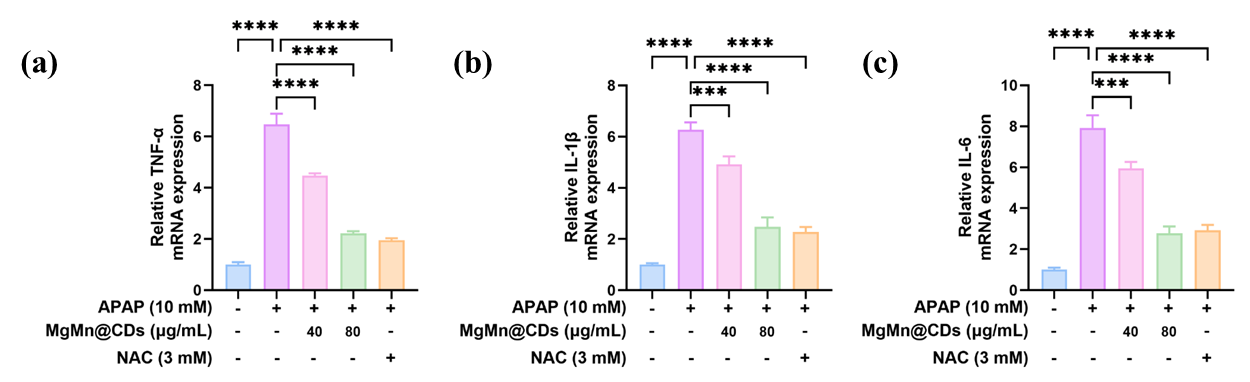


**Fig. S13.** Levels of mRNA expression of key proinflammatory cytokines: (**a**) TNF-α, (**b**) IL-1β, and (**c**) IL-6.


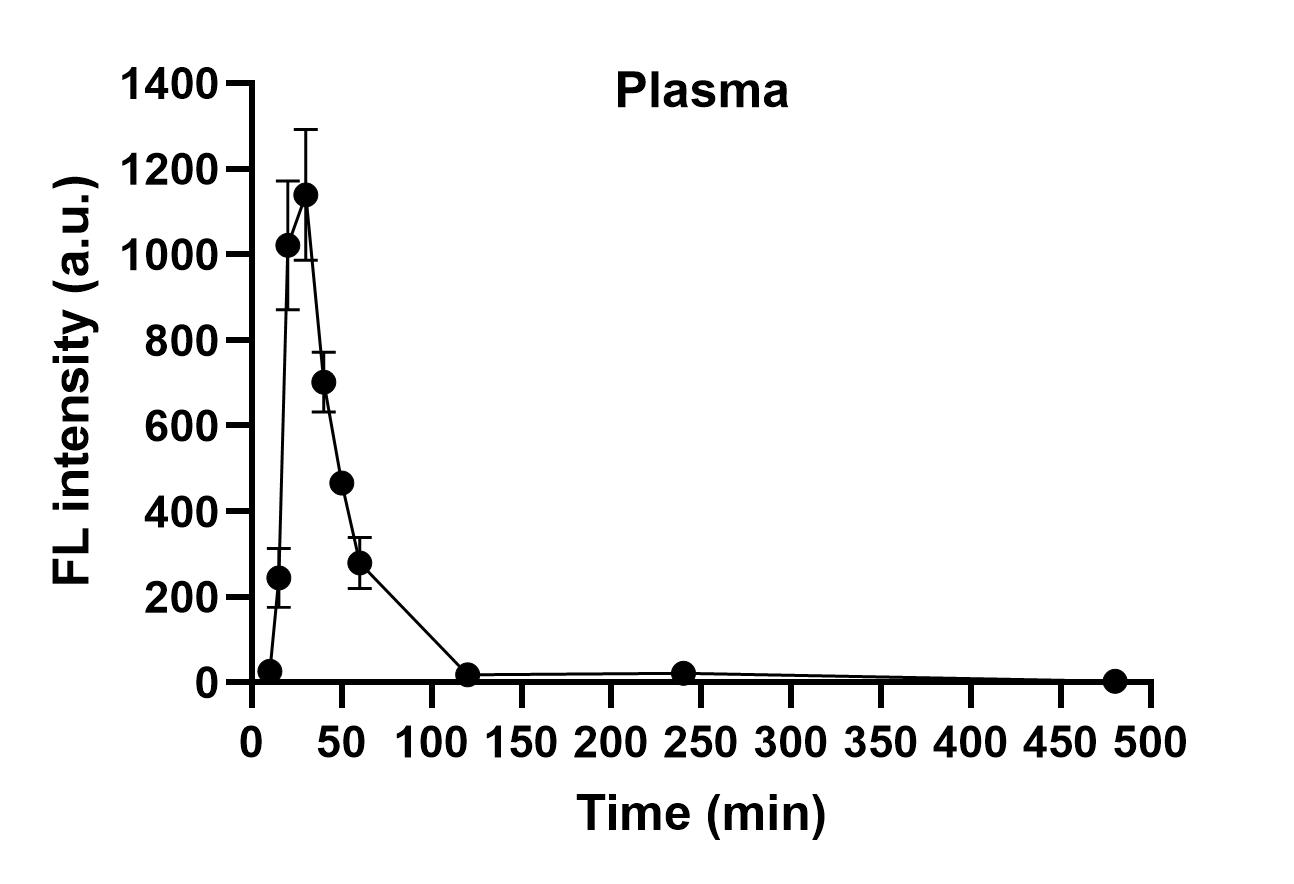


**Fig. S14.** *In vivo* pharmacokinetics of MgMn@CDs following i.p. administration.


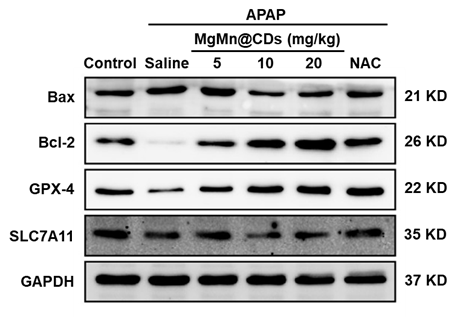


**Fig. S15.** Protein expression levels of key regulatory markers for apoptosis (Bax and Bcl-2) and ferroptosis (GPX4 and SLC7A11).

**Supplementary Tables**

**Table. S1** Metal contents in Mg-CDs, Mn-CDs and MgMn@CDs

| Sample | Content of Mg (wt %) | Content of Mn (wt %) |
| --- | --- | --- |
| Mg-CDs | 2.66 | - |
| Mn-CDs | - | 2.40 |
| MgMn@CDs | 2.09 | 1.86 |

**Table. S2** Average fluorescence lifetime and absolute quantum yields of CDs and MgMn@CDs.

| Sample | average fluorescence lifetime (ns) | absolute quantum yield (QY, %) |
| --- | --- | --- |
| CDs | 4.73 | 21.9 |
| MgMn@CDs | 3.14 | 16.4 |

**Table. S3** Fitting parameters of Mn K-edge EXAFS spectra of MgMn@CDs.

| Shell | N | R (Å) | σ^2^ (10^−2^ Å^2^) | ΔE_0_ (eV) | r-factor |
| --- | --- | --- | --- | --- | --- |
| Mn-N/O | 3.18 | 1.94 | 0.48 | 0.0052 | 0.04926 |
| Mn-C | 7.26 | 2.98 | 0.44 |  |  |
| Mn-N/O-C | 11.21 | 3.16 | 1.01 |  |  |

**Table. S4** Fitting parameters of Mg K-edge EXAFS spectra of MgMn@CDs.

| Shell | N | R (Å) | σ^2^ (10^−2^ Å^2^) | ΔE_0_ (eV) | r-factor |
| --- | --- | --- | --- | --- | --- |
| Mg-N/O | 3.62 | 2.17 | 0.32 | 3.208 | 0.0228 |
| Mg-C | 2.41 | 2.96 | 1.13 |  |  |
| Mg-N-Mg | 6.86 | 3.64 | 0.52 |  |  |

N is the coordination number for the absorber-backscatterer pair. R is the average absorber-backscatterer distance. σ^2^ is the Debye-Waller factor. ΔE_0_ is the inner potential correction. The accuracies of the above parameters are estimated as N, ±20%; R, ±1%; σ^2^, ± 20%; ΔE_0_, ±20%. The data range used for data ﬁtting in k-space (Δk) and R-space (ΔR) are 3.0–10.0 Å^−1^ and 1.0–3.0 Å, respectively.

S_0_^2^ is the amplitude reduction factor for the absorber-backscatterer pair. For Mn and Mg K-edge EXAFS spectra fitting, the S_0_^2^ value are 0.92 and 0.90, respectively.

**Table. S5** Comparison of MgMn@CDs with the CD-based SOD nanozymes.

| CDs nanozymes | Synthesis | Excitation/emission wavelength (nm) | Quantum yield (%) | SOD-like activity (U/mg) | Therapy | Ref |
| --- | --- | --- | --- | --- | --- | --- |
| T-Ag_SA_-CDs | hydrothermal method | 560/640 | / | 11814 (WST-1 by Dojindo) | Acute kidney injury | ^2^ |
| Cur@TB CD | hydrothermal method | / | / | 614.70 (WST-8 by Grace Biologicals) | Acute lung injury | ^3^ |
| Pt@CNDs | Oxidative etching method | / | / | 12605 (WST-1 by Dojindo) | PMA-induced ear inflammation | ^4^ |
| C-dots | Oxidative etching method | / | / | 9416 (WST-1 by Dojindo) | Diabetic wounds | ^5^ |
| C-dot SOD Nanozyme | Oxidative etching method | / | / | 10767 (WST-1 by Dojindo) | Neurological damage induced by ischemic stroke | ^6^ |
| Fluorescent C-dot SOD | hydrothermal method | 420/683 | 14% | 4049 (WST-1 by Dojindo) | Acute lung injury | ^7^ |
| Mg/Mn-CDs | hydrothermal method | 420/657 | 13% | 30864 (WST-1 by Dojindo) | Atopic dermatitis | ^1^ |
| MgMn@CDs | hydrothermal method | 420/657 | 16.4% | 20658 (WST-1 by Dojindo) | Acute liver injury | This work |

**Table. S6** Primers used for qRT-PCR.

|  | Primer sequences |
| --- | --- |
| Mouse GAPDH | F:5’ TGT GTC CGT CGT GGA TCT GA 3’ |
|  | R:5’ TTG CTG TTG AAG TCG CAG GAG 3’ |
| Mouse TNF-α | F:5’ AGGCTGCCCCGACTACGT 3’ |
|  | R:5’ GACTTTCTCCTGGTATGAGATAGCAAA 3’ |
| Mouse IL-6 | F:5’ ACA AGT CGG AGG CTT AAT TAC ACA T 3’ |
|  | R:5’ TTG CCA TTG CAC AAC TCT TTT C 3’ |
| Mouse IL-1β | F:5’ TCG CTC AGG GTC ACA AGA AA 3’ |
|  | R:5’ CAT CAG AGG CAA GGA GGA AAA C 3’ |

**References**

(1) Zhang Y, Shi J, Fang X, Li J, Fan X, Wang K, Gao G, Luo F, Xu N, Xia Z, et al. Mg/Mn co-doped carbon dot nanozyme with high superoxide dismutase-like activity and fluorescence for transdermal therapy of atopic dermatitis. Chem. Eng. J. 2025; 523: 168610.

(2) Tang T, Zhang J, Wang Y, Chen G, Yuan K, He Y, Li C, Hanif S, Yang Y, Wang Y, et al. Precision‐Engineered Silver Single‐Atom Carbon Dot Nanozymes for Theranostic Management of Acute Kidney Injury. Adv. Sci. 2026; 13 (16): e19393.

(3) Yang H, Qiu H, Shan J, Nie C, Lin Y, Shen Y, Wang X. Waste tobacco leaf-driven carbon dot nanozymes with high reactive oxygen species scavenging and anti-inflammatory activities for the amelioration of acute lung injury. Ind. Crops Prod. 2025; 229: 120958.

(4) Zhang Y, Gao W, Ma Y, Cheng L, Zhang L, Liu Q, Chen J, Zhao Y, Tu K, Zhang M, et al. Integrating Pt nanoparticles with carbon nanodots to achieve robust cascade superoxide dismutase-catalase nanozyme for antioxidant therapy. Nano Today. 2023; 49: 101768.

(5) Yan Z, Zhang Y, Chen Q, Li J, Ning X, Bai F, Wang Y, Liu X, Liu Y, Zhang M, et al. Carbon dot superoxide dismutase nanozyme enhances reactive oxygen species scavenging in diabetic skin wound repair. J. Adv. Res. 2025; 79: 691-706.

(6) Gao W, He J, Chen L, Meng X, Ma Y, Cheng L, Tu K, Gao X, Liu C, Zhang M, et al. Deciphering the catalytic mechanism of superoxide dismutase activity of carbon dot nanozyme. Nat. Commun. 2023; 14 (1): 160.

(7) Liu C, Fan W, Cheng WX, Gu Y, Chen Y, Zhou W, Yu XF, Chen M, Zhu M, Fan K, et al. Red Emissive Carbon Dot Superoxide Dismutase Nanozyme for Bioimaging and Ameliorating Acute Lung Injury. Adv. Funct. Mater. 2023; 33 (19): 2213856.
